# A neonatal nonhuman primate model of gestational Zika virus infection with evidence of microencephaly, seizures and cardiomyopathy

**DOI:** 10.1371/journal.pone.0227676

**Published:** 2020-01-14

**Authors:** Rosemary J. Steinbach, Nicole N. Haese, Jessica L. Smith, Lois M. A. Colgin, Rhonda P. MacAllister, Justin M. Greene, Christopher J. Parkins, J. Beth Kempton, Edward Porsov, Xiaojie Wang, Lauren M. Renner, Trevor J. McGill, Brandy L. Dozier, Craig N. Kreklywich, Takeshi F. Andoh, Marjorie R. Grafe, Heidi L. Pecoraro, Travis Hodge, Robert M. Friedman, Lisa A. Houser, Terry K. Morgan, Peter Stenzel, Jonathan R. Lindner, Robert L. Schelonka, Jonah B. Sacha, Victoria H. J. Roberts, Martha Neuringer, John V. Brigande, Christopher D. Kroenke, Antonio E. Frias, Anne D. Lewis, Meredith A. Kelleher, Alec J. Hirsch, Daniel Neal Streblow

**Affiliations:** 1 Division of Reproductive & Developmental Sciences, Oregon National Primate Research Center, Beaverton, Oregon, United States of America; 2 Vaccine & Gene Therapy Institute, Oregon Health & Science University, Beaverton, Oregon, United States of America; 3 Division of Pathobiology & Immunology, Oregon National Primate Research Center, Beaverton, Oregon, United States of America; 4 Division of Comparative Medicine, Pathology Services Unit, Oregon National Primate Research Center, Beaverton, Oregon, United States of America; 5 Division of Comparative Medicine, Clinical Medicine Unit, Oregon National Primate Research Center, Beaverton, Oregon, United States of America; 6 Department of Otolaryngology, Oregon Hearing Research Center, Oregon Health & Science University, Portland, Oregon, United States of America; 7 Advanced Imaging Research Center, Oregon Health & Science University, Portland, Oregon, United States of America; 8 Department of Neuroscience, Oregon National Primate Research Center, Beaverton, Oregon, United States of America; 9 Department of Ophthalmology, Casey Eye Institute, Oregon Health & Science University, Portland, Oregon, United States of America; 10 Department of Pathology, Oregon Health & Science University, Portland, Oregon, United States of America; 11 Veterinary Diagnostic Services Department, North Dakota State University, Fargo, North Dakota, United States of America; 12 Division of Comparative Medicine, Time Mated Breeding Services Unit, Oregon National Primate Research Center, Beaverton, Oregon, United States of America; 13 Division of Comparative Medicine, Behavioral Services Unit, Oregon National Primate Research Center, Beaverton, Oregon, United States of America; 14 Department of Obstetrics & Gynecology, Oregon Health & Science University, Portland, Oregon, United States of America; 15 Knight Cardiovascular Institute, Oregon Health & Science University, Portland, Oregon, United States of America; 16 Division of Neonatology, Department of Pediatrics, Oregon Health & Science University, Portland, Oregon, United States of America; Emory University School of Medicine, UNITED STATES

## Abstract

Zika virus infection during pregnancy is associated with miscarriage and with a broad spectrum of fetal and neonatal developmental abnormalities collectively known as congenital Zika syndrome (CZS). Symptomology of CZS includes malformations of the brain and skull, neurodevelopmental delay, seizures, joint contractures, hearing loss and visual impairment. Previous studies of Zika virus in pregnant rhesus macaques (*Macaca mulatta*) have described injury to the developing fetus and pregnancy loss, but neonatal outcomes following fetal Zika virus exposure have yet to be characterized in nonhuman primates. Herein we describe the presentation of rhesus macaque neonates with a spectrum of clinical outcomes, including one infant with CZS-like symptoms including cardiomyopathy, motor delay and seizure activity following maternal infection with Zika virus during the first trimester of pregnancy. Further characterization of this neonatal nonhuman primate model of gestational Zika virus infection will provide opportunities to evaluate the efficacy of pre- and postnatal therapeutics for gestational Zika virus infection and CZS.

## Introduction

Zika virus (ZIKV) is a mosquito-borne and sexually transmissible flavivirus endemic to central Africa and Southeast Asia, that has recently emerged in the Western Hemisphere. ZIKV infection in healthy adults is typically mild or asymptomatic, though more severe cases can result in ascending paralysis (Guillain-Barré syndrome) or inflammatory injury to the heart, brain or spinal cord [1, 2]. ZIKV infection in pregnant women increases the risk of miscarriage, preterm birth and congenital defects in affected infants. Fetal sequelae include microcephaly, neurological disorders, musculoskeletal abnormalities, auditory and visual impairment—symptomology collectively referred to congenital Zika syndrome (CZS) [3–5]. Potentially thousands of infants with CZS have been born to ZIKV exposed mothers since the beginning of the Western Hemisphere outbreak. Estimates of penetrance of adverse outcomes of congenital ZIKV infection have varied widely by region. In a cohort of 182 women with confirmed ZIKV infection in Rio de Janeiro, Brazil, adverse outcomes were observed in 46% of pregnancies [3]. In contrast, data collected from the US and US territories show an adverse outcome rate of ~4% (300/7056 completed pregnancies) between December 2015 and March 2018 [6]. Studies have confirmed an increased frequency of motor and cognitive delay in both microcephalic and non-microcephalic infants with *in utero* exposure to ZIKV [7–10]. Furthermore, congenital ZIKV infection is associated with ophthalmic abnormalities including focal pigmentary changes and chorioretinal scarring [11–13]. Functional assessment of vision in infants born with laboratory confirmation of ZIKV infection showed abnormalities in 92.6% of cases [14]. Sensorineural hearing loss, sleep disorders, and feeding complications including difficulty swallowing have also been recorded in children up to 2 years of age [9, 11, 15].

Following the 2015–16 epidemic throughout South and Central America, ZIKV has become newly endemic to the Caribbean and parts of the Americas suitable for year-round survival of the *Aedes* mosquito, the primary vector for ZIKV transmission [16, 17]. This expansion of endemicity has created a pressing need for the development of relevant preclinical CZS models to test vaccines and therapeutics directed against ZIKV infection and disease. Existing animal models have recapitulated much of the fetal pathology seen in human patients [18–28]. Studies in mice [29, 30] and pigs [31] have additionally reported restricted growth, seizures and behavioral and sensory abnormalities in neonatal animals following *in utero* exposure to Asian-lineage ZIKV. However, the complex neurological and behavioral aberrations observed in some human infants with CZS, such as deficiencies in fine motor and problem-solving skills, are difficult to assess in lower-phylogenetic order model organisms. Nonhuman primates (NHPs) are uniquely fit for studies of reproductive toxicity and teratogeny by virtue of their similarity to humans, especially with regard to reproductive and developmental biology [32]. ZIKV transmission across the hemochorial NHP placenta is well-documented [24, 28], with fetal injury and tissue tropism similar to that seen in humans [18–22, 33–35]. In this study, we sought to characterize the clinical and histopathological presentation of the rhesus macaque (*Macaca mulatta*, hereafter RM) neonate with *in utero* ZIKV exposure, with the aim of establishing a neonatal NHP model of gestational ZIKV infection. Five pregnant rhesus macaques were inoculated with ZIKV_PRVABC59_ between gestational day (GD) 53 and 55, near the end of the first trimester of pregnancy (term = 168 days) [36, 37]. Maternal viremia and fetal biometrics were tracked by serial biological fluid sampling and ultrasound, and clinical condition and neonatal development were assessed. We report a spectrum of clinical outcomes among the five ZIKV-exposed pregnancies, including one fetal and one early neonatal loss, one neonate with severe clinical abnormalities, and two apparently healthy infants. Histopathological findings correlated with the severity of clinical phenotypes, and were consistent with previous findings in fetal macaques and human neonatal cases of gestational ZIKV infection and CZS.

## Results

### Maternal ZIKV infection and immunity

**Fig 1:**
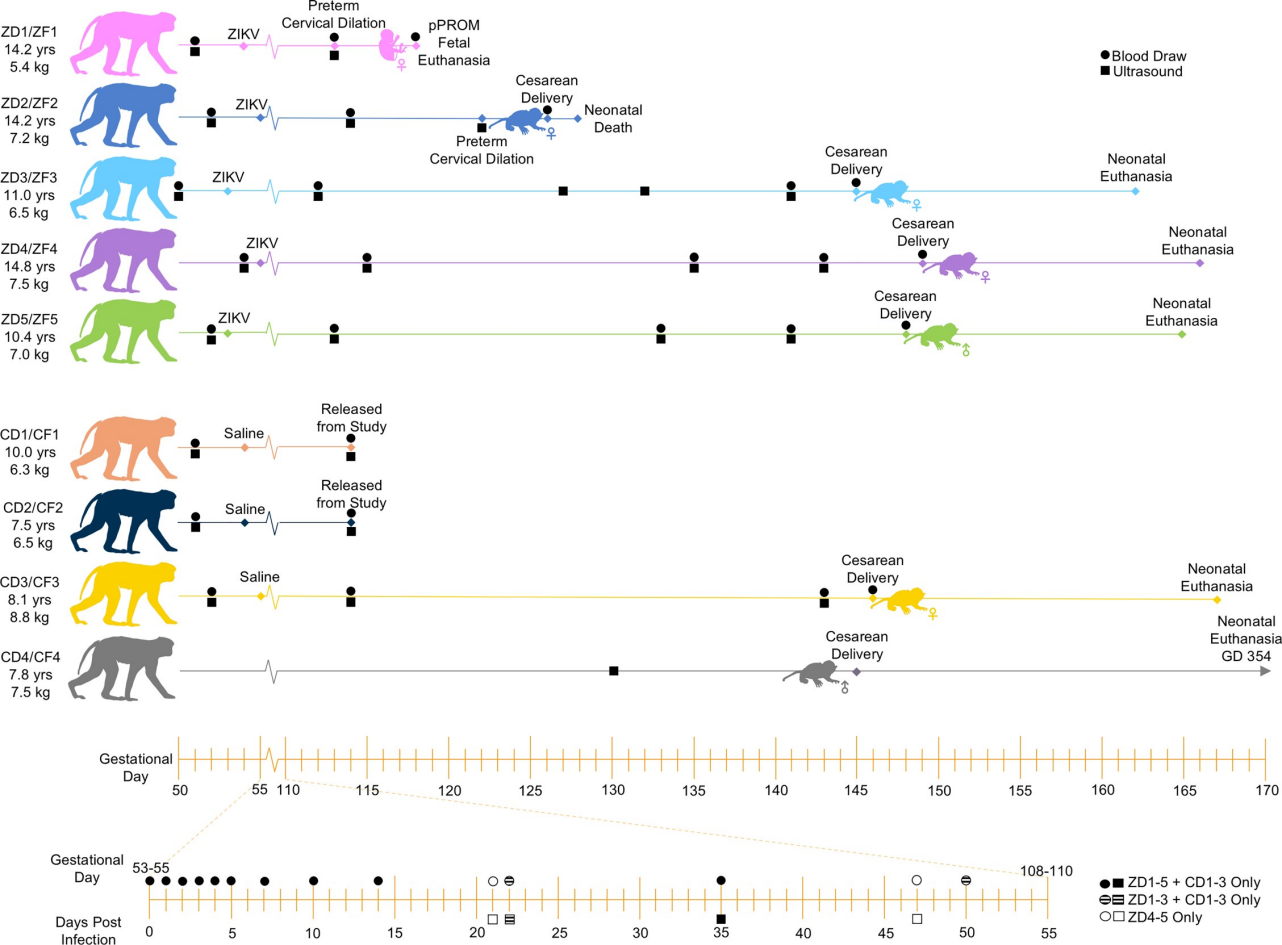
Cohort design and study timeline. Animal IDs denote experimental group (“Z” for ZIKV-infected, “C” for control), maternal/fetal identity (“D” for dam, “F” for fetus) and pregnancy number (1–5). For example: “ZF1” = ZIKV-infected fetus 1. Maternal age and weight listed in the left-hand column are at conception. Animal IDs and colors are consistent throughout all figures. Pregnant dams were infected subcutaneously with ZIKV_PRABC59_ on GD 53–55. Serial maternal blood draws and ultrasounds were performed at times shown. Dams were euthanized for tissue collection following fetal delivery by cesarean section. Neonates were survived for 2–22 days. Data from CD1-2 was limited to ultrasound biometrics and analysis of peripheral blood through GD 114.

**Fig 2:**
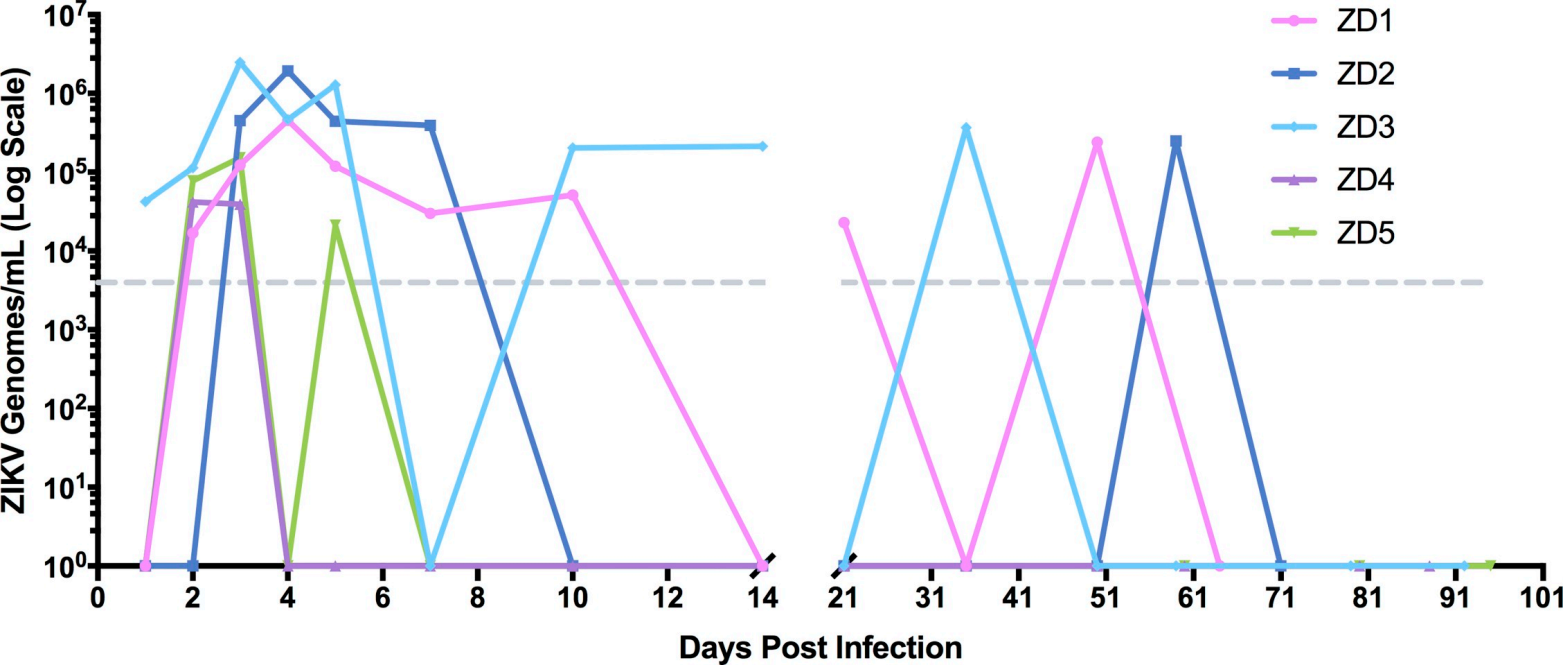
Maternal viremia. Total RNA was extracted from 250 μl of plasma. A one-step qRT-PCR assay was used to measure ZIKV RNA loads, reported as log_10_ ZIKV genomes/mL. The limit of detection (dotted grey line) was approximately 4,000 viral genomes/mL of plasma.

Five time-mated pregnant RM dams (ZD1-5) inoculated with Zika virus (ZIKV _PRVABC59_) and four control dams (CD1-4, vehicle injection) were monitored by routine physical exams, ultrasound, and serial peripheral blood sampling as shown in Fig 1. All infected dams developed mild bilateral axillary lymph node enlargement between 3 and 14 days post infection (DPI), but no rash or fever was observed. ZIKV RNA was detected in the peripheral blood plasma of all infected dams, with peak viremia occurring between 1–4 DPI (Fig 2). The length of viremia was variable; in ZD4 and ZD5, plasma ZIKV RNA levels were below the limit of detection by 7 DPI. The remaining three infected dams (ZD1-3) had evidence of plasma viral RNA (vRNA) detected sporadically until 30–70 DPI, consistent with previous publications [24, 28]. Longer viral persistence in ZD1-3 coincided with more severe pregnancy and neonatal outcomes in these animals (see below). All control dams were negative for viral RNA.

Maternal anti-ZIKV antibody responses developed between 7–14 DPI and peaked between 14–21 DPI (S1A Fig) in all Zika infected dams except ZD3. Anti-ZIKV antibody responses for ZD3 plateaued between 21–50 DPI, but then increased at 60–80 DPI. Flow cytometry to detect various CD8+ T cell lineages identified peak levels of proliferating maternal virus-specific MR1 Tet- Ki67+ CD8+ T cells at 7–14 DPI in ZIKV-infected dams (S2A Fig). Activated MR1 Tet- CD69+ CD8+ T cell numbers were also elevated between 2–7 DPI (S2B Fig).

ZIKV RNA was detected in tissues from all infected dams and fetuses/neonates following euthanasia between 64 and 112 DPI (Table 1), consistent with our previous studies in pregnant and non-pregnant ZIKV-infected RM [28, 38].

**Table 1:** Maternal and fetal/neonatal tissue viral loads.

	Animal ID	ZD1	ZF1	ZD2	ZF2	ZD3	ZF3	ZD4	ZF4	ZD5	ZF5
	GD of infection	54		55		53		55		53	
	Length of infection (d)	64	64	71	73	92	109	94	111	95	112
	Number of vRNA+ tissues (%)	8/48 (16.7)	9/39 (23.1)	28/38 (73.7)	30/57 (52.6)	6/60 (10.0)	9/39 (23.1)	3/55 (5.5)	5/40 (12.5)	12/23 (52.2)	7/40 (17.5)
lymphoid	Axillary LN	3.9	nd	2.7	nd	3.2	nd	nd	nd	nd	3
	Cervical LN			3.3	3	nd		nd		nd	nd
	Inguinal LN	nd	nd		3	nd	nd	nd	2.8	3.3	nd
	Mesenteric LN			5.4	nd	3.4	nd	nd	nd	2.8	nd
	Retroperitoneal LN	nd	nd	2.9	nd	nd	3.4	nd			
	Salivary LN	nd	nd		nd	2.7	nd	nd	nd		nd
	Spleen	nd	nd		nd	nd	nd	nd	nd	2.8	3.2
	Thymus	nd	nd	3.8	2.9			nd	nd		nd
	Tonsils	nd	3.9	2.9	3.6	nd	3				
cardio-pulmonary	Heart	nd	nd		3.1	nd	nd	nd	nd		nd
	Lung	nd	nd	nd	nd	nd	nd	nd	nd		nd
digestive	Gall bladder	nd	nd	2.8	2.9	nd	nd	nd	nd		nd
	Liver	nd	nd		3.1			nd	nd		nd
	Pancreas	nd		nd	nd	nd	nd	nd	nd		2.9
	Ileum			3.4	3	nd	nd				
	Duodenum				nd	nd					
	Jejunum			2.6	nd	nd					
	Colon			nd	nd	nd	nd				
	Esophagus				4.1	nd	nd				
	Stomach				3.1	nd	nd				
	Parotid salivary gland	nd	3.3	nd		nd		nd	nd		nd
	Submandibular gland	nd	4.3			nd		nd	nd	nd	nd
musculo-skeletal	Ankle	nd	nd	3.2	nd	nd	nd	nd		nd	nd
	Biceps	nd	nd	3.2	3.4	nd	nd	nd	nd	3	nd
	Brachioradialis	3.6	nd	3.6	3.2	nd		nd	3.1	2.7	nd
	Elbow	nd	nd	3.8	nd	nd	4.1	nd	nd		nd
	Finger	2.7	nd	3.7	3.4	nd	nd	3.4	nd	nd	nd
	Hamstring		nd	3		nd		2.8	nd		nd
	Knee	nd	nd	3.4	3.3	nd	nd	nd	nd	2.8	nd
	Quadriceps	nd	nd	5	2.7	nd	nd	nd	nd		3
	Soleus	nd	nd		nd	nd		nd	nd		3.1
	Toe	nd	nd	3.5	nd	nd	2.5	nd	nd		nd
	Triceps	nd	2.5	3.4		nd		nd	nd		nd
	Wrist	3.2	nd	2.7	3.1	nd	nd	nd			nd
integumentary	Skin	nd			nd	nd	nd	nd	nd		nd
	Mammary gland			nd		nd		nd			
genito-urinary	Bladder	nd	2.6		3.4	nd		nd	nd		nd
	Urethra				nd	3	nd	nd	nd	nd	
	Cervix	3.1	nd	nd	nd	nd	2.9	nd	2.8		
	Vagina	nd	nd	nd	nd	nd		nd	nd		
	Kidney	nd	nd	2.7	3	nd	nd	nd	nd		nd
	Ovary	nd	3.2		3.4	nd	nd	nd	nd	2.9	
	Fallopian tubes				nd		nd	2.7	nd		
	Uterus	nd	nd	2.8	3.5	nd	nd	nd	3		
	Testes										nd
nervous	Basal ganglion			nd	3.4	nd		nd		2.8	
	BR cerebellum				nd	nd		nd			
	BR frontal lobe	nd			2.9	nd		nd		nd	
	BR occipital lobe	nd		nd	nd	nd		nd		3	
	BR parietal lobe	nd			3	nd		nd		3	
	BR stem	nd			nd	nd		nd			
	BR temporal lobe	nd			nd	2.9		nd			
	Brachial plexus	nd	nd	5.2	nd	nd	2.6	nd		2.7	nd
	Dorsal root ganglion	nd		3.6	3.3	nd	nd	nd	nd		2.9
	Eye	nd			3.3	nd		nd	nd	nd	nd
	Femoral nerve	2.8	nd			nd		nd	3.2	2.6	3.5
	SC cervical	nd	nd	3.4	3.1	nd		nd			nd
	SC lumbar	2.8	2.7		nd	nd	3.6	nd	nd		nd
	SC thoracic	3	nd	3.7	3.2	nd	3.2	nd	nd		nd
	Sciatic nerve	nd	nd	nd	3.3	3.1	nd	nd	nd	nd	nd
	Trigeminal ganglion		2.7		3.5	nd	nd	nd		nd	
endocrine	Adrenal gland	nd			3.2	nd	nd	nd	nd		nd
	Pituitary gland	nd		3.2	nd	nd	2.8				
	Thyroid	nd	3.3	3.8	nd	nd	nd	nd	nd	nd	nd

Total RNA was isolated from tissues. ZIKV RNA levels were quantified using a one-step qRT-PCR assay and are presented as log_10_ viral copies per μg RNA. The limit of detection was approximately 400 genomes (2.6 log_10_) per μg of total RNA. Percent of vRNA+ tissues was calculated as a proportion of tested tissues only. The proportion of vRNA+ tissues in ZD5 may be skewed due to relative under-sampling. nd = virus not detected; grey cell = tissue not tested; GD = gestational day.

### Fetal biometrics

**Fig 3:**
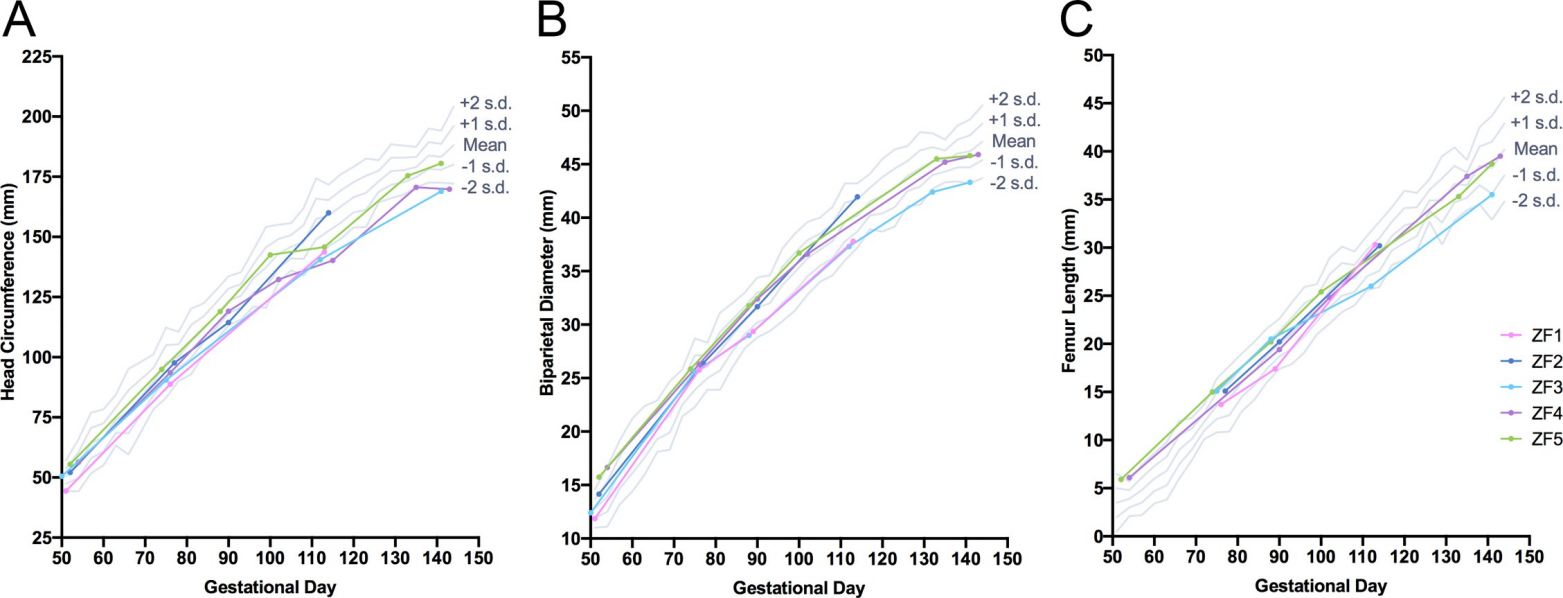
Fetal ultrasound biometrics. Serial ultrasound biometrics were measurements across gestation including (A) head circumference, (B) biparietal diameter and (C) femur length in the five ZIKV-infected fetuses. Reference ranges were plotted using data from Tarantal *[38]*.

Measurements of fetal head circumference, biparietal diameter and femur length are reported in Fig 3. While no major structural defects were noted by ultrasound in any of the animals, a sustained reduction in head circumference (≥ 2 s.d. from mean) was observed in ZF3 and ZF4 beginning at 60–61 DPI (GD 113, 112 and 115, respectively; Fig 3A) when compared to published fetal growth data from RMs [38]. Reduced biparietal diameter was also present in ZF3 beginning on GD 132 (Fig 3B). Femur length measurements were within 2 s.d. from the mean at all time points for all animals, though femur length in ZF3 was at least 1 s.d. below the mean from 59 DPI until delivery (Fig 3C).

### Pregnancy outcomes following ZIKV infection

Pregnancy outcomes are summarized in Table 2. Zika-infected infants were delivered between GD 118 and 148, with complications including premature rupture of membranes and cervical ripening necessitating preterm emergency cesarean delivery of ZF1 and ZF2 at GD 118 and 124, respectively. Due to concerns of late-pregnancy complications and based on placental abnormalities on ultrasound, infants ZF3-5 were delivered preterm by scheduled C-section between GD 145–148. CF3 and CF4 were delivered by C-section on GD 145–146 as age-matched controls for ZF3-5, as shown in Table 2.

**Table 2:** Summary of clinical and necropsy findings.

Fetal ID	ZF1	ZF2	ZF3	ZF4	ZF5	CF3	CF4
Dam ID	ZD1	ZD2	ZD3	ZD4	ZD5	CD3	CD4
Inoculum	ZIKV	ZIKV	ZIKV	ZIKV	ZIKV	Saline	None
GD of Inoculation	54	55	53	55	53	55	N/A
GD of Delivery	118	126	145	149	148	146	145
GD (PD) of Demise	118 (0)	128 (2)	162 (17)	166 (17)	165 (17)	167 (21)	354 (209)
Pregnancy and Neonatal Outcome	Early cervical ripening, pPROM, emergency hysterotomy with fetal euthanasia	Early cervical ripening, clinically indicated hysterotomy, neonatal death (septicemia)	Elective hysterotomy, live birth, clinically indicated euthanasia	Elective hysterotomy, live birth, experimental euthanasia	Elective hysterotomy, live birth, experimental euthanasia	Elective hyster-otomy, live birth, experimental euthanasia	Elective hyster-otomy, live birth, experimental euthanasia
Maternal Utero-placental Pathology	Small mono-discoidal placenta; multifocal infarctions, hemorrhage, villous stromal mineralization and perivasculitis	Endometritis and myometritis; plasmacytic deciduitis; villous stromal mineralization	Multifocal infarctions, acute and chronic hemorrhage, villous fibrin deposition and stromal mineralization	Multifocal infarctions, villous fibrin deposition and mineralization, neutrophilic and plasmacytic deciduitis, decidual leukocytoclastic vasculitis	Focal infarction, villous fibrin deposition and mineralization, neutrophilic and plasmacytic deciduitis, mild decidual leuko-cytoclastic vasculitis	NP	Rare stromal mineralization, focal infarction
Neonatal Clinical Presentation	N/A	Prolonged hypoxemia during resuscitation, hyperglycemia, hypotonia, bradycardia, apnea, cardio-respiratory arrest	Seizures, tachypnea, cardiomegaly, axial hypotonia, lower limb weakness, dysphagia, retarded growth	Possible mild auditory impairment complicated by presence of earwax	NSF	NSF	NSF
Fetal Gross Pathology	NSF	Cerebral meningeal and ventricular hemorrhage secondary to sepsis; small cerebellum; chin dermatitis	Cardiomegaly with mild dilation of right and left ventricles; reduced brain mass	NSF	NSF	NSF	NSF
Fetal CNS Lesions	**Cerebrum**—Multifocal periventricular mineralization; ependymal degeneration; increased apoptosis of neuroprogenitor cell population; increased cells noted in molecular layer of one section, presumed immature neurons (GFAP-/Iba1-/CD45-); increased astrocytes (GFAP+) in molecular layer of cingulate gyrus **Cerebellum**–Moderate decrease in Purkinje cell population **Spinal cord**—Mild to marked rarefaction and edema of white matter, thoracic cord is more affected	Multifocal hemorrhage, edema and necrosis secondary to sepsis. Pathologies atypical to sepsis cannot be conclusively attributed to Zika virus infection: **Cerebrum**—Periventricular mineralization, increased apoptosis of neuroprogenitor cells **Cerebellum**–Minimal decrease in Purkinje cell population, decreased density of external granular layer	**Cerebrum—**Ventricular dilation, mild ependymal loss; fewer neuroprogenitor cells **Cerebellum**—Attenuation of the external granular cell layer; moderate decrease in Purkinje cell population **Spinal cord—**Glial necrosis, white matter, minimal in lumbar cord	**Cerebrum**—Unilateral periventricular ependymal cell degeneration and loss, focally extensive **Cerebellum**—Multifocal, minimal decrease in Purkinje cell population	**Cerebellum**—Multifocal, moderate decrease in Purkinje cell population and decrease in external granular cell layer **Spinal cord—**Swollen myelin sheaths, rare gitter cells and solitary spheroid in cervical cord **Dorsal root ganglion—**Scattered, minimal lymphocytic infiltrates	NSF	No gross lesions; microscopic lesions not assessed
Fetal Lesions in Other Tissues	**Urinary bladder**—Multifocal, minimal histiocytic infiltrates	**Skin of chin**—Focal, mild, neutrophilic dermatitis **Biceps brachii, left—**Hemorrhage and edema secondary to sepsis **Brachioradialis, right**—Hemorrhage and edema secondary to sepsis	**Heart**—Cardiomyopathy, left ventricle, increased cardiomyofiber diameter, cardiomyocyte karyomegaly	**Heart—**Ventricle, cardiomyocyte hypertrophy and karyomegaly, rare	**Heart—**Multifocal, mild to moderate cardiomyocyte vacuolar degeneration and multifocal, mild karyomegaly	NSF	NSF
Fetal Post-mortem MRI	NP	T1W and T2W lesions secondary to sepsis	Reduced brain volume and surface area	NSF	NSF	NSF	NP

NSF = no significant findings, NP = not performed/not assessed, N/A = not applicable.

### Placental pathology

**Fig 4:**
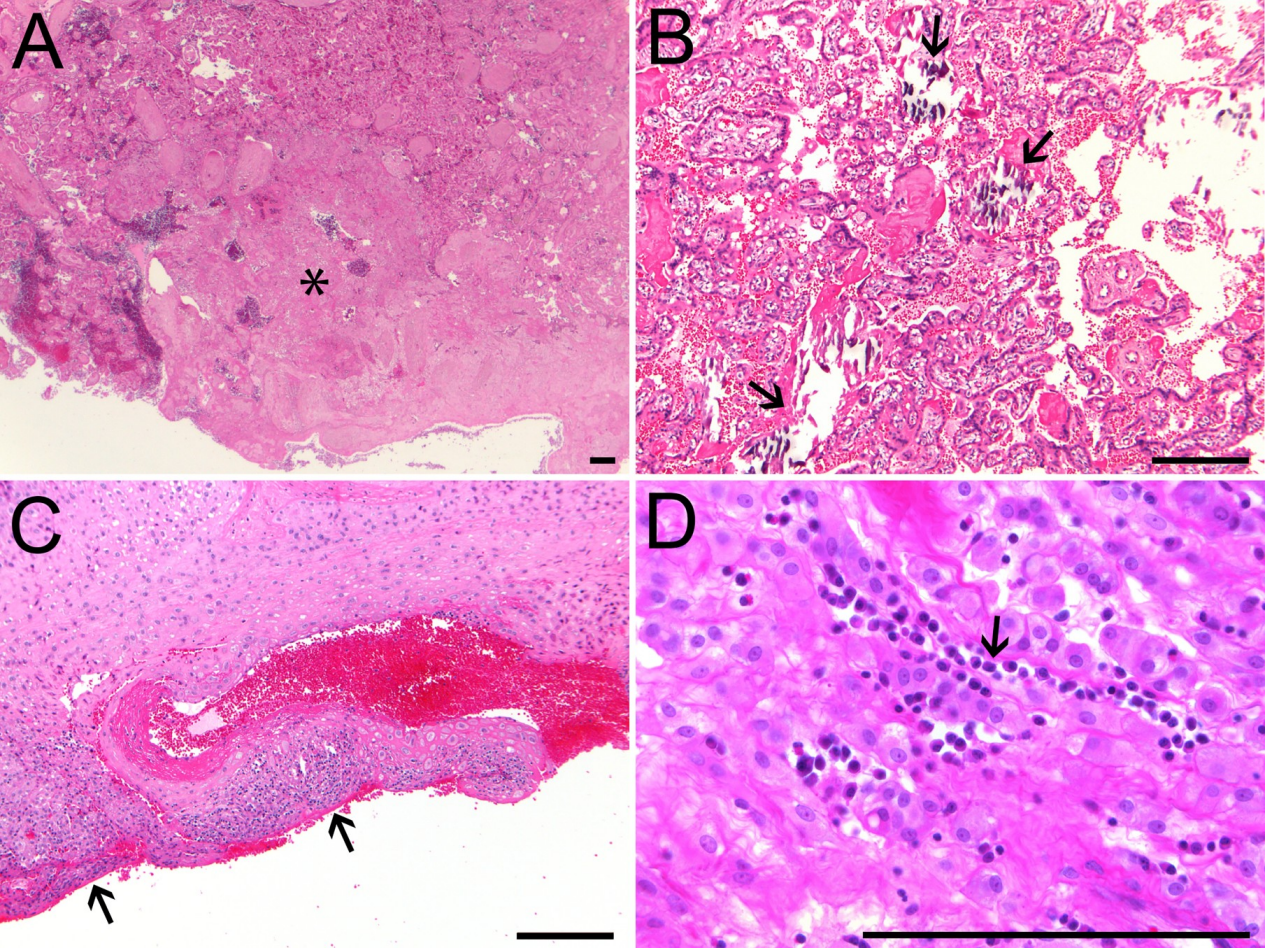
Placental histopathology of ZIKV-infected RM. All five placentas from ZIKV-infected animals showed histological evidence of pathology. Representative images are presented of (A) placental infarction (asterisk) in ZF3; (B) villous stromal mineralization (arrows) in ZF5; (C) lymphocytic and plasmacytic leukocytoclastic vasculitis (arrows) in the decidua of ZD4; and (D) chronic plasmacytic inflammation in the decidua of ZD2 (arrow). Scale bars = 200 μm except where otherwise noted.

Histopathological assessment of placentas from ZIKV-infected pregnancies identified signs of inflammation and injury including multifocal infarctions, hemorrhage, vasculitis, deciduitis and stromal mineralization (Fig 4). In addition to the pathology noted in other placentas from ZIKV-infected pregnancies, the placenta of ZD1 was small and mono-discoidal (rather than the more common bi-discoidal structure in RM placentas), and ZF1 placental cultures were positive for *Staphylococcus aureus* and *Staphylococcus epidermidis*. However, culture of the amniotic fluid in ZD1 was negative and there was no histological evidence of chorioamnionitis or funisitis (Table 2).

In comparison, the placentas of control pregnancies had minimal signs of inflammation. A focal infarction and rare stromal mineralization noted in the placenta of CD4 were within the normal range of histological findings in third trimester RM placentas.

### Postnatal clinical outcomes

Pregnancy outcomes, clinical and histological findings are detailed in Table 2. ZF1 was euthanized at delivery (GD 118) based on early gestational age; resuscitation of this infant was not attempted. ZF2 received intensive clinical care following delivery on GD 126, but did not survive past 42 hours. Early-onset neonatal sepsis caused by *Staphylococcus aureus* was identified as the likely cause of death; bacterial cultures from ZF2 brain were positive for S. aureus, while placental and maternal uterine cultures were negative. Resuscitation of ZF3-5 and CF3-4 was uncomplicated. Newborn Health Assessment (modified Apgar) scores for these five animals were within normal limits (5–8 at delivery and 8–12 within 30 minutes) for monkeys delivered at approximately 90% of term gestation (S3C Fig) [39]. Detailed neonatal vital signs and required respiratory support are displayed in S3 Fig. All infected and control infants initially required respiratory support in the form of positive pressure ventilation (PPV), continuous positive airway pressure (CPAP) and supplemental oxygen. Neonatal assessments were performed on the remaining Zika-infected infants (ZF3-5) before euthanasia and tissue collection on postnatal day (PD) 17. Following the initial neonatal transition and resuscitation, ZF4-5 and CF3-4 remained relatively stable throughout the entire neonatal period. Conversely, ZF3 was affected with severe CZS-like symptomology including seizures and cardiomyopathy.

### Cardiac pathology

**Fig 5:**
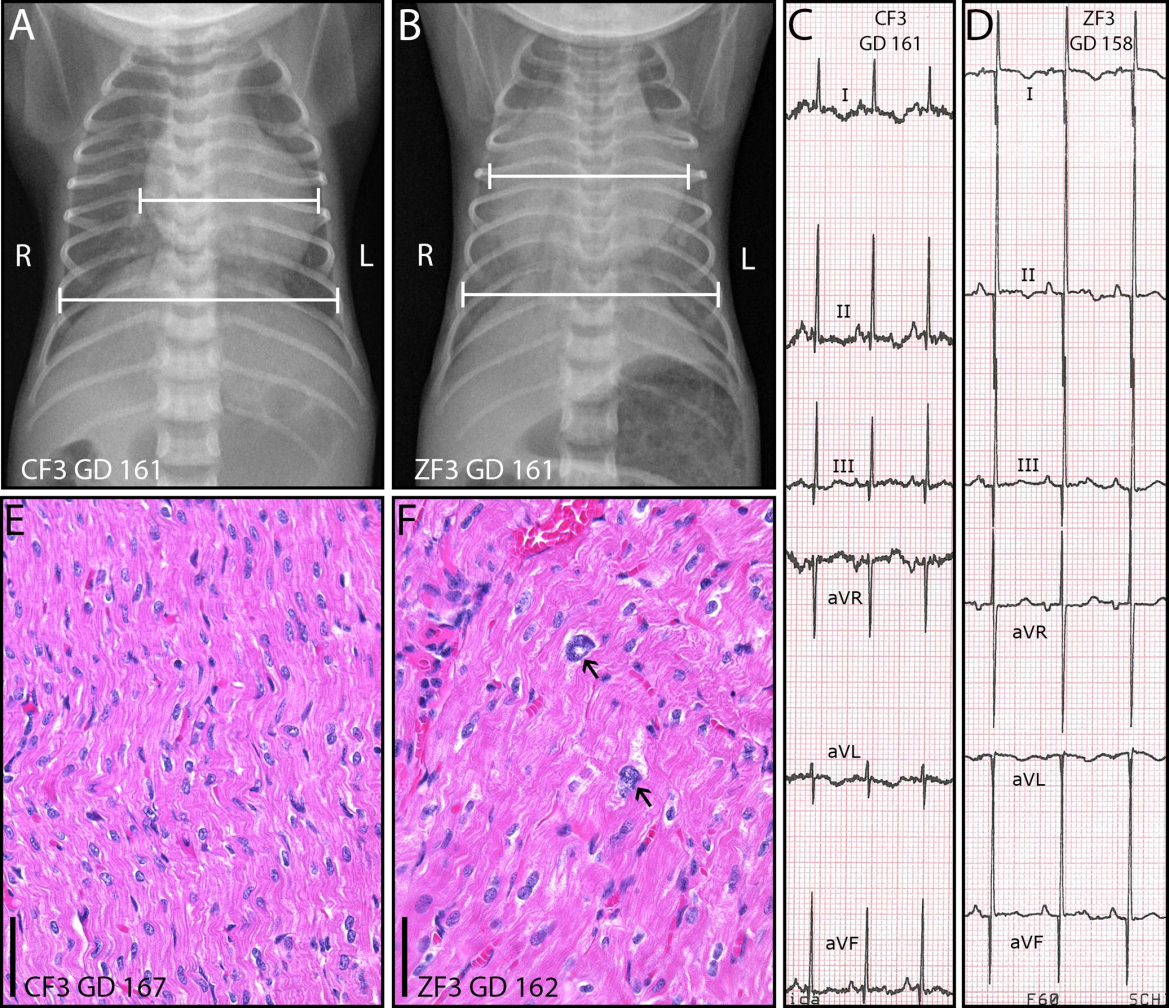
Cardiomyopathy in ZIKV-infected infant ZF3. (A-B) GD 161 ventrodorsal thoracic radiographs showing cardiomegaly in infected infant ZF3 (B, CTR 0.77) relative to control CF3 (A, CTR 0.63). (C-D) Limb lead only electrocardiograms of (C) control infant CF3 (GD 161) and (D) ZIKV-infected infant ZF3 (GD 158); scale 10 mm/mV, 50 mm/s. Increased R and Q wave voltages (R_amp_, Q_amp_) in ZF3 limb leads II (R_amp_ 3.7 mV, Q_amp_ 1.8 mV) and III (R_amp_ 2.5 mV, Q_amp_ 0.8 mV) suggest left ventricular enlargement. (E-F) Hematoxylin and eosin staining of the myocardium with myocyte karyomegaly (arrows) and cytomegaly in ZIKV-infected infant ZF3 (F) relative to control CF3 (E). Scale bars = 50 μm.

Major cardiac abnormalities became evident in ZF3 on PD 10, when the infant presented with sustained tachypnea (S3E Fig). A 4/6 systolic heart murmur was detected upon auscultation and thoracic radiographs revealed cardiomegaly (cardiothoracic ratio [CTR] 0.73) that was not present on PD 1 (CTR 0.47) or in age-matched control CF3 (Fig 5A). A limb lead only electrocardiogram (ECG) performed on PD 14 was suggestive of left ventricular hypertrophy with increased voltages in leads II and III (Fig 5D) [40–42]. Lead II R wave amplitude (R_amp_) was 85% higher in ZF3 in than in CF3 (3.7 vs 2.0 mV). A limited echocardiogram was suggestive of mild pericardial effusion but revealed no evidence of patent ductus arteriosus nor any structural defect, and was insufficient to diagnose or rule out left ventricular hypertrophy. Measures to treat suspected congestive heart failure (furosemide, 1–2 mg/kg IV BID) were not effective. Based on this clinical presentation, including persistent tachypnea and progressive cardiac enlargement (CTR 0.77 on PD 16, Fig 5B), ZF3 was euthanized on PD 17 (GD 162, 109 DPI). On gross examination of the heart, there was a mild pericardial effusion and mild dilation of the right and left ventricles, but no apparent septal or valvular defects. Microbial culture of the pericardial fluid was negative. Heart weight (4.60 g) for ZF3 was 3.87 s.d. above the mean for historical age-matched fetal controls (GD 161–162, n = 13; range 2.29 g– 3.86 g, mean ± s.d.: 2.86 ± 0.45 g, S4F Fig) [43]. Microscopic evaluation of the myocardium revealed cardiomyocyte karyomegaly (Fig 5F) and increased cardiomyofiber diameter in the left ventricular free wall compared to the right ventricular free wall. These histological changes in ZF3 are consistent with left ventricular hypertrophy; rapid progression of cardiac enlargement and gross dilation of the ventricles suggests a dilated cardiomyopathy. There was no evidence of inflammatory infiltrates to suggest active myocarditis, and heart tissue from ZF3 was negative for ZIKV RNA at 109 DPI (Table 1). Although ZF4 and ZF5 also had rare hypertrophic and karyomegalic cardiomyocytes (Table 2), these animals had no clinical signs of heart disease. Comprehensive echocardiograms of both ZF4 and ZF5 showed patent ductus arteriosus (PDA) without evidence of right ventricular enlargement or dysfunction on PD 0–1; PDA was considered normal for gestational age (GD 148–150), and was resolved at follow-up on PD 16–17 (near term equivalency). ECGs in ZF4 and ZF5 were unremarkable relative to CF3. Cardiothoracic ratio at PD 1 for ZF3 and at all time points for ZF4-5 and CF3-4 was within the normal range (0.46–0.67) published by Wozniak et al. for very low birth weight, appropriate for gestational age (preterm) human neonates [44]. No other cardiac abnormalities were noted in the remaining control or Zika-infected infants.

### Functional neurological abnormalities

Infants displayed a range of neurodevelopmental phenotypes and pathologies, from seizure activity and motor delay (ZF3) to apparently normal development for gestational age (ZF4 and ZF5). Infant ZF3 exhibited seizure activity soon after delivery at GD 145, which continued throughout the neonatal period and was refractory to antiepileptic therapy (phenobarbital and levetiracetam; S5 Fig). Seizures were characterized by unresponsiveness to tactile stimulation; nystagmoid eye movements; oral-buccal-lingual movements (chewing, grimacing or tongue protrusion); rhythmic twitching of the face, scalp and limbs; and bicycling motions in the limbs, all consistent with “subtle” neonatal seizure symptomology as described by Volpe *et al*. [45]. An electroencephalogram (EEG) to assess epileptiform activity in ZF3 could not be obtained prior to clinically-necessary euthanasia on PD 17 (GD 162). No obvious seizure activity was observed in other infected or control infants, and no epileptiform activity was observed during sleeping EEGs performed on ZF4 and ZF5.

Basic motor, reflex and sensorimotor development was assessed using the Infant Behavioral Assessment Scale (IBAS) [46], with supplementary assessments of righting behavior and resistance to passive movement in the limbs. Infant ZF3 was again the most severely affected, presenting with axial hypotonia, hindlimb weakness, lethargy and poor motor coordination. At the time of euthanasia (PD 17), infant ZF3 had achieved criteria on only 2/6 (33.3%) assessments of motor development, versus 6/6 (100%) for age-matched controls CF3 and CF4 (S6A and S6C Fig). Delays in motor development in ZF3 were attributable to hindlimb weakness. When awake ZF3 was vocal, responsive, able to turn over and briefly lift her head and upper body; however, she did not obtain normal motor milestones such as sitting, locomotion and self-feeding that were evident in all other neonates (CF3-4 and ZF4-5). Feeding difficulties including abnormal swallowing reflex (dysphagia) in ZF3 potentially contributed to reduced feed volumes and poor postnatal growth in this infant (S7 Fig). In contrast, infected infants ZF4 and ZF5 had appropriate postnatal weight gain and met criteria for most IBAS assessments prior to euthanasia, demonstrating apparently normal motor and reflex development when compared to similarly nursery-reared age-matched controls (S6 Fig).

### Sensory pathology

Sensory screening was not performed in ZF3 due to clinical instability; however, results from retinal imaging and auditory testing were collected for ZF4-5 (S8 and S9 Figs). Ultra-widefield retinal images were unremarkable in infants ZF4 and ZF5 and did not reveal any obvious abnormalities in the peripheral retina (S8A and S8B Fig). No abnormalities were observed in retinal layer thickness measured by OCT (S8C and S8D Fig).

Auditory brainstem response (ABR) thresholds were detected in ZF5 at all frequencies tested and were consistent with the average thresholds from 1-month-old control infants (n = 3). No ABR thresholds were detected in ZF4 at 2 and 8 kHz, and thresholds at 12, 16, and 26 kHz were elevated relative to controls, suggesting hearing impairment (S9A Fig). However, a buildup of cerumen adjacent to the tympanic membrane in ZF4 may have contributed to the elevated thresholds observed. Distortion product otoacoustic emissions (DPOAE) were detected in response to 60 dB SPL stimuli in ZF4 at 12 kHz only, suggesting suboptimal outer hair cell function. ZF5 produced DPOAEs at all frequencies tested except at 4 kHz, which was consistent with responses in controls. Cell-counts performed in ZF4-5 cochleae immunostained with the hair cell marker myosin VIIA revealed no outer hair cell loss and consistent inner hair cell numbers throughout the length of the cochlea (S9E Fig). Hair cell innervation was assessed by whole mount neurofilament immunofluorescence and showed a normal distribution pattern of radial fibers (S9C and S9D Fig), consistent with hair cell sparing in hearing-impaired ZIKV-infected mice [30].

### Postmortem neuropathology

Brain weights at necropsy were similar for ZF4-5 and control infants. However, ZF3 had a whole brain weight (44.2 g) 2.9 s.d. below the mean for age-matched historical Oregon National Primate Research Center (ONPRC) controls (GD 161–162, n = 13; range 50.7 g– 60.3 g, mean ± s.d.: 55.6 ± 3.9 g, S4E Fig) [43]. Additionally, the brain weight of ZF2 (34.0 g at GD 128) was 2.0 s.d. below the mean (41.25 ± s.d. 3.57 g, n = 9) for GD 125 RM controls from Kerr *et al*. [47] (S4E Fig), despite normal measurements of head circumference and biparietal diameter on ultrasound (Fig 3A and 3B).

**Fig 6:**
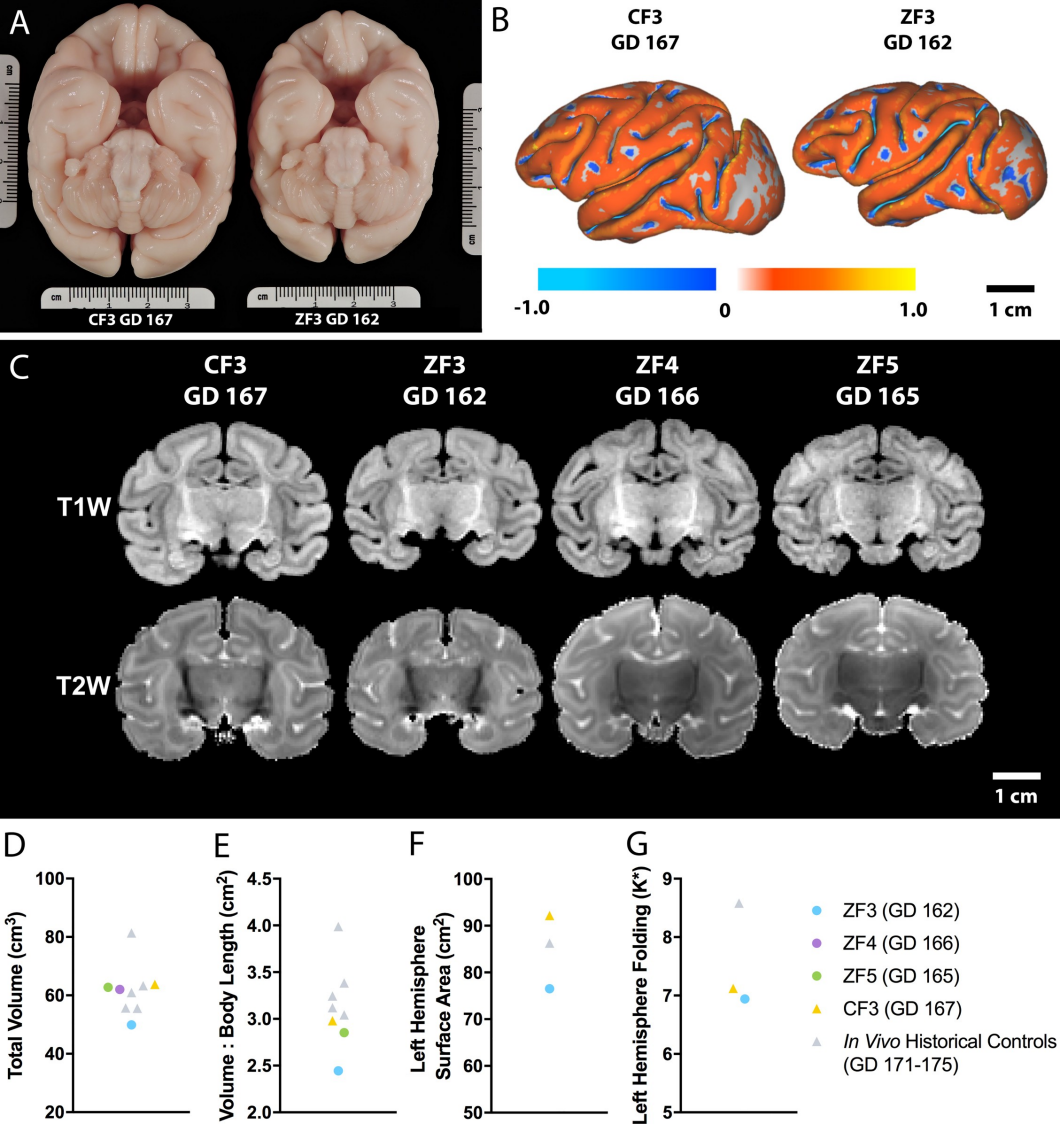
Postmortem MRI analysis of neonatal brains. (A) An inferior view of the fixed brains of CF3 (left, GD 167) and ZF3 (right, GD 162) shows a grossly smaller brain in the infected animal. (B) Three-dimensional cortical surface models were generated to assess cortical surface area and folding. (C) No lesions or apparent structural abnormalities were detected on T1W or T2W images of CF3 and ZF3-5. (D) Total brain volume was reduced in ZF3 relative to CF3 and *in vivo* controls [49]. (E) Total brain volume normalized to crown-rump length was also reduced in ZF3. (F) Left hemisphere surface area was reduced in ZF3 relative to CF3 and an *in vivo* ONPRC historical control. (G) Left hemisphere nondimensionalized mean curvature (K*) as a measure of folding in ZF3 was not appreciably different from CF3.

Postmortem MRI scans were conducted on fixed brains from ZF2-5 and CF3, with images, brain volume and cortical folding shown in Fig 6. Multifocal T1-weighted (T1W) hyperintense and T2-weighted (T2W) hypointense MRI lesions in ZF2 were attributed to intracerebral hemorrhaging following septicemia [48]. No lesions were detected on postmortem brain MRI of ZF3-5 or CF3. A 3D cortical surface model was generated from ZF3 and CF3 to assess relative surface area and curvature (Fig 6B). There was reduced brain volume (Fig 6D and 6E) and surface area (Fig 6F) in ZF3 relative to CD3, to an *in vivo* ONPRC historical control, and to *in vivo* controls from Kelleher *et al*. scanned at GD 171–175 [49]. Nondimensionalized mean curvature (K*) as a measure of cortical folding was not appreciably different between ZF3 and CF3 (Fig 6G). Brain volume in ZF4 and ZF5 was unremarkable relative to controls.

**Fig 7:**
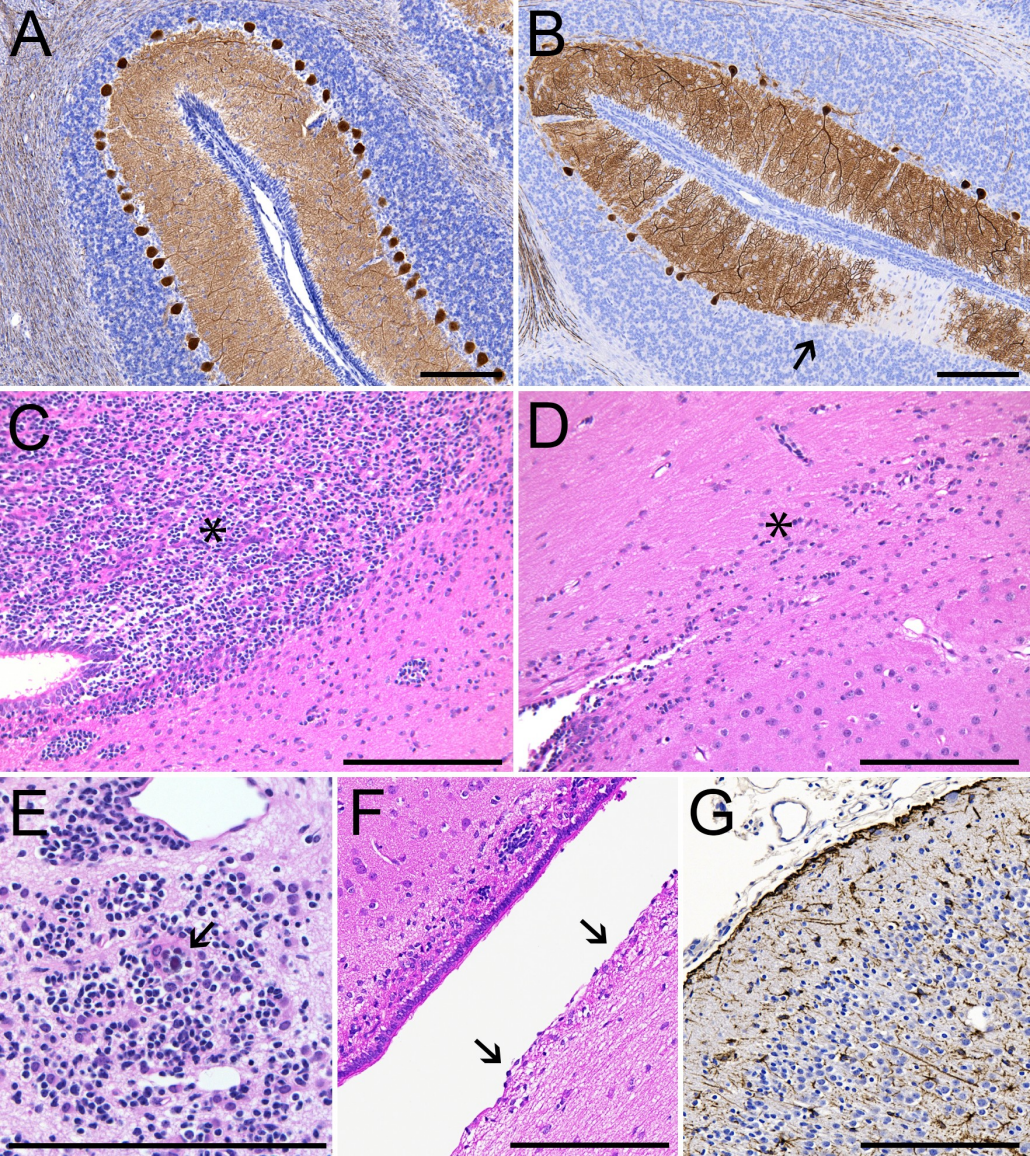
Fetal and neonatal neuropathology. Brain tissues from all five ZIKV-infected animals showed histological evidence of pathology. Representative images are shown in Panels A-G. (A) Calbindin staining (brown) showing normal distribution and morphology of Purkinje cells in the cerebellum of CF3, GD 167. (B) Relative paucity of Purkinje cells (arrow) in the cerebellum of ZF3, GD 162. (C) Hematoxylin & eosin staining showing neural progenitor cells (NPCs) in the periventricular white matter of CF3 (asterisk). (D) Relative paucity of NPCs in the periventricular white matter of ZF3 (asterisk). (E) Focal mineralization (arrow) among a cluster of NPCs in ZF1 cerebrum (GD 118). (F) Unilateral ependymal erosion (arrows) of the lateral ventricle in ZF4, GD 166. (G) GFAP staining (brown) of reactive astrocytes in the cingulate gyrus of ZF1, GD 118. Scale bars = 200 μm.

Histopathological assessment of the brains of Zika-infected neonates identified cerebral abnormalities (Table 2) including mineralization, ventricular ependymal degeneration, increased numbers of reactive astrocytes and loss of neuroprogenitor cells (Fig 7C–7G). Ventricular dilation was also noted in ZF3, the only neonate in the cohort with clear neurological symptoms. Spinal cord white matter injury and inflammation were noted in ZF1 and ZF3. In the cerebellum, Purkinje cell populations were decreased 34–40% in ZF3 and ZF5 relative to CF3, with a milder 7% decrease in ZF4 (Fig 7A and 7B, S1 Table). Histological analysis of ZF2 was complicated by neonatal septicemia.

## Discussion

In this study of gestational Zika virus infection in rhesus macaques we have identified a range of pregnancy complications, placental injury, and postnatal neurological and cardiac abnormalities. We also report the first case of microencephaly in a neonatal rhesus monkey exposed to Zika virus infection *in utero*. The spectrum of outcomes observed in this NHP model, from fetal loss and severe postnatal neurological impairment to apparently phenotypically normal neonates, recapitulates the range of perinatal complications that have been reported in clinical studies of congenital Zika virus syndrome since the emergence of ZIKV in Brazil from 2014–2016. We observed 100% vertical transmission in this cohort of pregnant dams infected by subcutaneous injection with a clinically relevant strain and dose of ZIKV with viral RNA detectable in all neonates. We also observed that those infants with the most severe phenotypic presentation were exposed to extended maternal viremia during gestation, whereas infants with more normal postnatal outcomes were born from pregnancies with a shortened viremic period and with lower overall viral loads. The observation that maternal viremic status may be associated with postnatal outcomes, coupled with our ability to comprehensively assess placental function *in utero* [28], maternal infection and immune status, as well as postnatal functional sequelae of infection, creates the opportunity to further our understanding of gestational ZIKV infection and CZS in an animal model with extensive translational similarities to humans.

While microcephaly was the first recognized symptom of CZS, the disorder is now understood to be a complex syndrome with multi-organ pathology and a range in severity of outcomes. Among the three ZIKV-infected neonates, one infant presented with multiple serious clinical abnormalities including seizures, feeding difficulty, cardiomyopathy, reduced brain size, hindlimb weakness and poor motor coordination relative to control animals. These pathologies are rare in the general RM population. Since 2014, there have been 4034 live births in the RM breeding colonies at ONPRC. In this time there have been 28 reported cases (0.7%) of any neurological abnormality in an infant <1 year old, including seizures and motor disturbances, and 6 recorded cardiac abnormalities (0.1%). There have been no cases of comorbid neurological and cardiac defects, and no cases of microcephaly/microencephaly in colony neonates.

Seizures are a common phenotype in human infants with suspected CZS [9, 15, 50–52], and seizure-like activity has been reported in piglets with *in utero* exposure to ZIKV [31]. Additionally, delayed development of motor skills and reflexes has been reported in human neonates born to ZIKV-infected mothers [7–10]. Fetal cardiac abnormalities are a less well-characterized risk of ZIKV infection in pregnancy. Three echocardiographic studies have reported three- to ten-fold higher prevalence of major cardiac structural defects, including ventricular hypertrophy and dilation, in infants with presumed CZS compared to infants in the general population [53–55]. An additional case report describes an infant with presumed CZS who presented with a clinical phenotype similar to ZIKV-infected NHP infant ZF3: dysphagia, axial hypotonia, motor delay and a congenital heart defect [56]. Mild pericardial effusion and subjective cardiac enlargement on ultrasound have been reported previously in two RM fetuses with gestational ZIKV infection [21]. Following numerous case reports in adult patients, ZIKV has joined the group of viruses implicated in causing myocarditis that includes parvovirus B19, influenza, coxsackievirus B3, rabies virus, rubella virus, cytomegalovirus, chikungunya virus, as well as other flaviviruses such as yellow fever virus, dengue virus and hepatitis C virus [57–60]. Viral induced myocardial tissue damage generates immunopathologic mediated alterations that can lead to cardiomyopathy and even, if severe enough, heart failure [60–62]. Damage to cardiac tissues may persist and progress to dilated cardiomyopathy even after clearance of vRNA and resolution of inflammatory processes, as we observed in ZF3 [61]. Furthermore, placental insufficiency has been implicated as a cause of congenital heart disease; placental dysfunction following gestational ZIKV infection may mediate the risk of heart defects in the fetus [28, 63]. These cardiac findings may have important implications for the management of human infants exposed to gestational ZIKV infection.

The hallmark finding in severe cases of CZS in humans is microcephaly/microencephaly, defined respectively as an occipitofrontal head circumference ≥2–3 s.d smaller than the mean for sex/age/ethnicity, and a brain weight ≥2 s.d. smaller than the mean [64]. In this study, three of the ZIKV-infected fetuses had reduced head circumference and/or biparietal diameter on third trimester ultrasounds. In ZF2 and ZF3 respectively, brain mass at euthanasia was 2.0 s.d. and 2.9 s.d. below the mean for age-matched controls. However, observation of reduced biparietal diameter and head circumference by ultrasound was predictive of small brain mass only in the most severely affected neonate, which is a potential limitation of antenatal imaging in identifying subtle brain changes in NHPs. Microencephaly has been reported in murine and porcine models of gestational ZIKV infection [25, 65], along with reduced growth velocity of fetal head circumference and biparietal diameter in previous NHP models [19, 20, 24]. This study is the first to confirm that microencephaly and associated functional neurological deficits can occur in rhesus monkeys following *in utero* ZIKV exposure, which is a potentially translationally relevant finding for the development of vaccines and interventional strategies to prevent the long-term morbidities associated with Zika virus infection and poor neurodevelopmental outcomes.

Neuropathological findings in this cohort of ZIKV-infected infants were consistent with findings in human infants with CZS. Ependymal erosion [5, 19, 21], cerebral mineralization [66–68] and NPC loss [18, 66, 69] have all been previously reported in humans and fetal NHPs following *in utero* ZIKV exposure. Purkinje cell infection and degeneration have been previously reported in murine models of neonatal ZIKV infection [70, 71]. In ZF2 and ZF3 with histopathological evidence of loss of NPCs throughout the cerebrum, this finding coincided with reduced brain mass, which is consistent with hypotheses that NPC degeneration may be partly responsible for the characteristic microcephaly seen in severe CZS cases [25, 72]; however, we note that ZF2 was affected by acute septicemia, and NPC loss in this infant cannot conclusively be attributed to ZIKV infection. Perinatal brain injury warrants further investigation in this model in order to fully characterize the range of neuropathological changes associated with congenital ZIKV infection.

Proposed hypotheses suggest that prolonged viremia in pregnant women may be caused by and/or serve as an indicator of active fetal infection [73–76]. As stated above, we observed 100% vertical transmission in pregnant dams infected subcutaneously with a clinically relevant strain and dose of ZIKV. Dams ZD1-3 all had incidence of late infection viremia (>21 DPI); prolonged viremia is a finding in ZIKV-infected rhesus monkeys that is apparent during pregnancy but less common in non-pregnant monkeys [73, 75, 77]. Despite robust anti-ZIKV specific antibody responses in both the maternal and fetal/neonatal circulation, ZIKV RNA was still detected in tissues from all animals. Notably, the three animals with extended viremia were the same animals with the most severe adverse fetal/neonatal outcomes. Conversely ZD4-5 had no detectable viremia after 7 DPI, lower tissue vRNA loads, lower proportions of ZIKV+ fetal tissues, and also had no obvious adverse pregnancy or postnatal complications. However, we do note that the shortened gestation/postnatal period for ZF1-2 limit our ability to compare tissue viral loads between all 5 animals. In this preliminary study, small sample size precludes a robust analysis of the correlation between viral loads and fetal disease; however, our data are consistent with such an effect.

Premature cervical ripening and pPROM were also observed in ZIKV-infected dams in the current study. Epidemiological and clinical data have linked ZIKV infection during gestation with adverse pregnancy outcomes, including stillbirth and fetal loss [3, 78–81]. Pregnancy loss is also consistent with outcomes in prior studies of first trimester ZIKV_PRVABC59_ infection in pregnant rhesus monkeys [21]. Premature delivery is a complicating factor in the analysis of ZIKV pathology in this cohort; this outcome may be related to infection, and was necessitated in this translational model for the clinical management of each pregnancy. Furthermore, placental insufficiency in ZIKV-infected pregnancies has been implicated as a contributing factor in adverse pregnancy outcomes, including preterm birth and fetal mortality [3, 27, 28, 72]. Placental pathology in this cohort mirrored results from prior studies: spiral artery vasculitis, deciduitis, multiple infarctions, hemorrhage and mineralization have all been previously reported in human and macaque pregnancies with ZIKV infection [23, 28, 34, 82, 83]. pPROM in the case of ZD1 may have been complicated by bacterial infection, although this infection was likely secondary to pPROM given lack of evidence of chorioamnionitis. We have previously reported robust fetal proinflammatory responses to gestational ZIKV infection [28]; viral-induced inflammatory reactions during pregnancy can result in aberrant fetal immune development, potentially contributing to perinatal vulnerability to infection [84, 85].

In this study, we have demonstrated that fetal/neonatal rhesus monkeys exposed to ZIKV during the first trimester *in utero* display a range of clinical phenotypes, including hallmark symptoms of human CZS. Despite the inherent limitations of nonhuman primate studies, the advantages of establishing a model of gestational ZIKV infection and CZS in rhesus monkeys, including similarities to humans in placentation, fetal and neonatal neurodevelopment, immune function and ZIKV pathogenesis make this model uniquely valuable for enhancing our knowledge of the pathogenesis and effect of gestational ZIKV infection on postnatal development and long-term health. The main goal of these ongoing studies is the development of novel vaccines and therapies to prevent and treat gestational ZIKV infection and CZS.

## Materials and methods

### Ethics statement

All Zika virus infection experiments utilizing nonhuman primates were performed in compliance with guidelines established by the Animal Welfare Act for laboratory animal housing and care and in accordance with Oregon National Primate Research Center (ONPRC) Institutional Animal Care and Use Committee approved animal protocol (IACUC #1099). NHP studies were performed in ABSL-2 containment facilities at the ONPRC. The ONPRC is accredited by the Association for Assessment and Accreditation of Laboratory Animal Care (AAALAC) International. Experimental procedures were overseen by veterinary staff, and were designed to minimize potential animal distress, pain and discomfort. Animals were pair-housed whenever possible to promote social behavior, and were fed a standard species-appropriate diet adjusted for age and body weight. Intake was monitored and animals received food supplements and other enrichment devices. All animals were monitored by research and veterinary staff for criteria of humane endpoints, which included any condition resulting in untreatable pain or distress, or a poor prognosis for survival to the end of the study period. At the time points listed in the manuscript, animals were euthanized according to the recommendations of the American Veterinary Medical Association 2013 Panel on Euthanasia.

### Experimental design

Study design is shown in Fig 1. On GD 53–55, time-mated pregnant macaques ZD1-ZD5 were subcutaneously inoculated with 10^5^ focus forming units (ffu) of ZIKV_PRVABC59_ as previously described [28]. CD1-3 were subcutaneously inoculated with 1 mL normal saline on GD 54–55. CD4 was not inoculated. Dams were evaluated daily for clinical signs of disease. Serial rectal temperature measurement, peripheral blood draws and fetal/placental ultrasound analyses were performed under ketamine sedation (10 mg/kg, IM) as detailed in Fig 1. Manual cervical exams of ZD1-3 and CD1-3 were performed by a maternal-fetal medicine specialist as previously described [21]. Dexamethasone (0.25 mg/kg PO BID) was administered to ZD2-5 and CD3 24 hours prior to delivery to encourage maturation of the fetal lungs. Dams ZD2 and ZD3 additionally received indomethacin (25 mg PO BID) for 2 (ZD3) or 3 (ZD2) days prior to delivery to delay labor until a scheduled cesarean section could be performed. Pregnancies were delivered by hysterotomy under ketamine (10 mg/kg, IM), propofol (0.3–1 mg/kg IV) and isoflurane (1.25%) anesthesia. pPROM was observed in ZD1 on GD 118, necessitating early delivery of ZF1 by cesarean section on the same day. Due to extreme prematurity, ZF1 was euthanized immediately following delivery. ZD2 exhibited preterm cervical dilation on GD 122; ZF2 was delivered on GD 126 and survived for approximately 42 hours under intensive clinical care, but succumbed to sudden cardiopulmonary arrest later attributed to early-onset sepsis. ZF3, ZF4 and ZF5 were delivered on GD 145, 149 and 148, respectively. ZF3 reached a humane endpoint on postnatal day 17, with a poor prognosis following progressive cardiac enlargement, and was clinically euthanized the same day. ZF4 and ZF5 were survived for 17 days before experimental euthanasia. Infected dams were euthanized after delivery and tissues were collected to determine viral persistence and dissemination. Uninfected controls CF3 and CF4 were delivered on GD 148 and 145 respectively. CD1 and CD2 were released from the study on GD 114; data from these pregnancies is restricted to flow cytometric analysis of maternal peripheral blood.

Fetal resuscitations were attended by a board-certified specialist in neonatal-perinatal medicine (R.L.S.). Respiratory support was provided immediately after delivery as previously described [49], following a standardized algorithm for neonatal resuscitation [86]. Briefly, airways were suctioned and CPAP (5 cm/H_2_O) or PPV (peak inspiratory pressure 20–30 cm/H2O and positive end-expiratory pressure 5 cm/H_2_O) with blended oxygen initiated with a Neo-Tee^®^ Infant T-piece Resuscitator (Mercury Medical). Infants ZF2, ZF3 and CF3 were intubated and surfactant (100 mg phospholipids/kg, Survanta, AbbVie Inc.) was administered intratracheally; infants ZF4, ZF5 and CF4 did not require intubation or surfactant. PPV, CPAP and/or flow by oxygen support were continued as needed for all infants to maintain SpO2 >93%. Modified APGAR scores were assessed at 1, 5 and 30 minutes after birth using a metric developed for neonatal NHPs by Ruppenthal *et al*. [39]. Intravenous access was established through the distal cephalic vein with a peripheral IV catheter (Introcan Safety IV Catheter, 22G × 1″, Teflon Straight, Braun Medical Inc.). Parenteral nutrition was administered as previously described [49] using a central catheter (First PICC^™^ S/L 26GA, 1.9F, Argon Medical Devices) inserted into the saphenous vein. Infants were monitored 24 hours a day during the study period by research staff trained in nonhuman primate handling and neonatal care, with multiple daily evaluations by a veterinarian. Thoracic radiographs were taken on PD 1 to confirm proper PICC placement for all neonates except ZF2. Additional radiographs were taken of ZF3 (PD 12 and 16), ZF4 (PD 8 and 17), ZF5 (PD 7 and 17) and CF3 (PD 14 and 17). Cardiothoracic ratio was calculated using the C/T2 method described by Edwards, Higgins and Gilpin [87], with thoracic width measured at the inner aspect of the 8^th^ rib. Infants were housed in incubators (Hill-Rom Air-Shields Isolette C2000; "Pet"iatric Supply Intensive Care Unit) with blankets and soft enrichment devices for the duration of the study. Enteral feeding was introduced on PD 1 with donated human breast milk (Northwest Mothers Milk Bank, 0.5–1 mL every 2–4 hours), with volumes increasing throughout the neonatal period depending on the infant’s appetite and tolerance. Formula (Similac^®^ for Supplementation, Abbott Nutrition) was introduced gradually beginning on PD 4–8; introduction of formula was delayed to PD 12 in ZF4 because of transient diarrhea and agitation during breast milk feedings. Offered milk and formula volumes were restricted to prevent overfeeding. PICC lines were removed in all infants between PD 6 and 9, once oral feed volumes exceeded 120 mL/kg/day.

Infant reflex, motor and sensorimotor development was assessed as previously described [49] and scored using the NHP Infant Behavioral Assessment Scale published by Sackett et al. [46] with additional assessments of righting behavior and resistance to passive movement. Due to heightened biosafety protocols used for work with ZIKV-infected animals, investigators were not blinded to treatment group. Assessments were separated by at least two days. All neonates except ZF2 were assessed three times by PD 7, and 1 (ZF4, ZF5) or 3 (ZF3, CF3, CF4) more times before euthanasia on PD 17–22. Testing on ZF4 and ZF5 occurred at least 24 hours after administration of sedative for auditory or visual exams.

Infected dams (ZD1-5) and infant ZF1 were euthanized and taken to necropsy immediately following delivery. Infant ZF2 was necropsied following death on PD 2; infants ZF3-5 and CD3 were euthanized on PD 17–22 as indicated in Fig 1. The brain was mechanically perfused with saline followed by 4% paraformaldehyde via cannulation of the common carotid artery. Perfused whole brains were post-fixed in 4% paraformaldehyde for 72 hours and transferred to 0.01M phosphate buffered saline (PBS) for postmortem MRI. Portions of tissues (joints, muscles, organs, brain, spinal cord, eyes, glands, lymph nodes and bone marrow) and fluid samples (amniotic fluid, blood, cerebral spinal fluid, and urine) from these animals were collected and stored in RNAlater (tissue storage, Qiagen), Trizol (RNA isolation; ThermoFisher) and medium (virus isolation; ThermoFisher); tissues were also fixed in 10% buffered formalin and processed for paraffin IHC and MRI. Whole blood was centrifuged over lymphocyte separation medium (Corning) for 30 minutes at 1,459 x g to isolate peripheral blood mononuclear cells (PBMCs) and plasma and then analyzed for lymphocyte phenotype and frequency by flow cytometry. Plasma vRNA levels were determined by qRT-PCR and anti-ZIKV antibody levels were quantified by ELISA.

Except where otherwise indicated, historical control data is previously-unpublished archived data collected from healthy rhesus macaque pregnancies at ONPRC between 2007 and 2019. In some cases, other data from these control pregnancies has been previously published [43, 88, 89]. Housing, husbandry, veterinary care and fetal/neonatal euthanasia of historical control animals were consistent with care provided to the experimental cohort in this paper, described above. Experimental interventions during historical control pregnancies were limited to sedated ultrasound, MRI and/or blood draws; subcutaneous implantation of miniosmotic pumps containing a vehicle control; low-dose vitamin C supplementation; and procedures occurring immediately prior to fetal delivery and euthanasia with no anticipated effect on fetal organ weights or measures. ONPRC also maintains RM breeding colonies in large, outdoor-housed social groupings. Animals living in the breeding colonies are dam-reared and not assigned to any experimental protocol; they are fed a standard species-appropriate diet with frequent enrichment, and receive regular veterinary care. Data on the frequency of various pathologies in breeding colony infants is included as the best available representation of the general RM population.

### Zika virus preparation

ZIKV_PRVABC59_ was obtained from the CDC and passaged twice in C6/36 cells (American Type Culture Collection (ATCC)) as previously described [28, 90]. Supernatant from infected C6/36 tissue culture was concentrated through a 20% sorbitol cushion and titrated in Vero cells (ATCC) through a focus-formation assay. The viral inoculum was sequenced as previously described [28, 90].

### Phenotypic analysis of maternal peripheral blood mononuclear cells

Flow cytometry was performed as previously described [28] to quantify maternal immune cell phenotypes. Approximately 1×10^6^ PBMC were placed in 50–100μl of RPMI-10 media for tetramer staining with 50nM dasatinib. MR1-tetramer was added at a final concentration of 100nM and cells were incubated in the dark at room temperature for 1hr. Surface staining antibodies were then added, and cells incubated for an additional 30 min in the dark at room temperature. Cells were then washed once with 1 × PBS and fixed with the fix/perm reagent from the eBioscience Foxp3/Transcription Factor Staining Kit for 1hr at room temperature in the dark (Thermo Fisher Scientific, Waltham, MA). Cells were then washed twice with the Foxp3/Transcription Factor Staining Kit Permeabilization Reagent. Intracellular antibodies were added and incubated for 1hr at room temperature in the dark. Cells were washed twice with permeabilization reagent, resuspended in 200μl 2% PFA, and stored at 4°C until data acquisition. The antibodies used in this study included: anti-CD3 (clone: SP34-2, Pacific Blue; BD Biosciences), anti-CD8 (clone: SK1, TruRed; BD Biosciences), anti-CD4 (clone: L200, Amcyan; BD Biosciences), anti-CD28 (clone: CD28.2, PE; BD Biosciences), anti-CD95 (clone: DX2, FITC; BD Biosciences), anti-CD69 (clone: FN50, ECD; Biolegend, San Diego, CA), anti-Ki-67 (clone: B56, FITC; BD Biosciences), anti-GzmB (clone: GB12, PE; Life Technologies, Carlsbad, CA), anti-perforin(clone: Pf-344, FITC, Mabtech, Sweden), anti-Bcl-2 (clone: Bcl-2/100; BD Biosciences), and LIVE/DEAD Fixable Yellow Dead Cell Stain (Life Technologies, Carlsbad, CA) was used to assess cell viability. MR1-tetramer was prepared as previously described [91]. Phenotyping was performed using an LSRII instrument (BD bioscience) and the data was analyzed with FlowJo Software (TreeStar).

### Anti-ZIKV Enzyme-Linked Immunosorbent Assay (ELISA)

Anti-ZIKV antibody levels were measured in maternal and fetal blood plasma by end point dilution ELISA as previously described [28]. Briefly, high-binding polystyrene 96-well plates (Corning) were coated with purified ZIKV particle preparations overnight at 4°C. The plates were blocked with PBS containing 2% milk and 0.05% Tween (ELISA-Block) for 1 hr at room temperature. Plates were washed with ELISA-Wash and incubated with two-fold dilutions of plasma samples in ELISA-Block for 2 hrs. Plates were washed with ELISA-Wash and then incubated for 30 mins with secondary anti-monkey IgM or IgG (Rockland, Inc.) conjugated with horseradish peroxidase. Bound secondary antibody was detected using the OPD substrate (Life Technologies) followed by addition of HCl to stop the reaction. The plates were read using a Synergy HTX Microplate Reader (BioTek) at 490nm. Endpoint antibody binding titers were calculated by Log/Log transformation and analyzed using GraphPad Prism v6 software. The limit of detection was a dilution = 1:50.

### ZIKV qRT-PCR analysis

Total RNA from tissues, blood, urine, and cerebrospinal fluid (CSF) was isolated as previously described [28] using TRIzol (Invitrogen). ZIKV RNA levels were measured by a one-step quantitative real time reverse transcription polymerase chain reaction assay (qRT-PCR) using TaqMan One-Step RT-PCR Master Mix (Applied Biosystems). A sample containing 250ng of total RNA from tissue samples or 1/10th volume of RNA isolated from 250 μl of liquid samples was used in each reaction. Based on a lower detection limit of 100 genomes per reaction [28, 90], the limit of detection of the assay is 400 genomes/ug of total RNA from tissue, and 4,000 genomes/mL of plasma. Primers and probes were as follows: Forward: 5’-TGCTCCCACCACTTCAACAA (ZIKV_PRVABC59_ genome sequence nucleotides 9797–9816); Reverse: 5’-GGCAGGGAACCACAATGG; (complement of nucleotides 9840–9857); and TaqMan probe: 5’ Fam-TCCATCTCAAGGACGG-MGB (nucleotides 9819–9834). Forward and reverse primers were used at 250 nM in the reaction, and the probe at 200 nM. For RNA standards, RNA was isolated from purified, titered stock of ZIKV_PRVABC59_. RNA yield was quantified by spectrometry and the data was used to calculate genomes/μl. Focus-forming units (ffu)/μl was calculated based on titer of stock. ZIKV RNA was serially diluted 1:10 into Vero cell RNA (25 ng/μl) and amplified in triplicate using primers and conditions described previously [28].

### Histological analysis

Following MRI, paraformaldehyde-fixed brains and formalin-fixed maternal, fetal and placental tissues were paraffin-embedded, sectioned at 5 μm and stained with hematoxylin & eosin (H&E). All sections were reviewed by veterinarians board-certified in anatomic pathology (L.M.A.C, A.D.L. and H.L.P.). Cardiac sections from ZF3 and CF3 were additionally reviewed by an MD anatomic pathologist with clinical interests in pediatric pathology and cardiovascular pathology (P.S.); brain sections from ZF1-5 and CF3 were reviewed by an MD anatomic pathologist board-certified in neuropathology (M.R.G.); and placental tissue sections from ZF1-5 and CF3 were reviewed by an MD board certified pathologist (T.K.M.). A limited set of tissues were selected for immunohistochemical staining; paraffin-embedded serial tissue sections from the brain were deparaffinized in Histoclear II (National Diagnostics HS-202), rehydrated in graded ethanol and stained using the VECTASTAIN^®^ Elite^®^ ABC HRP Kit (Vector Laboratories PK-6100). CNS sections were incubated for 1 hour at room temperature with primary antibodies against GFAP (1:1000 Millipore AB5804), Iba1 (1:500 Wako 019–19741) and CD45 (1:200 Cell Signaling CS 13917S), or for 24 hours at 5°C with primary antibodies against calbindin D28-K (1:2000 Swant 300). Antigen retrieval with heated pH 6.0 sodium citrate was performed prior to CD45 and calbindin staining.

For Purkinje cell counts, the central cerebellar folia from twelve different sites of each case including four gyri, four sulci and four sides were examined at 400x. Purkinje cells with visible nuclei were counted in one field of view. Purkinje cell numbers of ZF3-5 were compared to those of CF3. Purkinje cell numbers of ZF1-2 were compared to a GD 127 ONPRC historical control.

For imaging of the middle and inner ear structures, decalcified skull bases from ZF4 and ZF5 were prepared for immunofluorescent staining as follows. The otic capsule was opened and cochlear turns were dissected from the inner ear. The lateral wall was trimmed, and tectorial membrane removed prior to immunostaining. Hair cells and neuronal filaments were detected with rabbit anti-myosin 7a (1:200 Proteus 25–6790) and chicken anti-neurofilament (1:10000 Abcam ab4680), respectively. Secondary antisera were goat anti-rabbit Alexa-Fluor 488 (1:200 Invitrogen A32731) and goat anti-chicken Alexa-Fluor 647 (1:200 Invitrogen A21449). Whole mount cochlea were imaged on an Olympus FV1000 using a z-stack of 3 μm. Optical sections were imported into Imaris 9.1 (Bitplane, South Windsor, CT, USA) for image analysis. Hair cell counts were generated by spot detection within a specified distance from the filament drawn along the basilar membrane and verified by visual analysis of the 3-D reconstruction.

### Echocardiography

Clinical veterinarians performed a basic echocardiogram on infant ZF3 (PD 15). A parasternal long axis view was obtained using a GE LOGIQ E9 to evaluate the left ventricle and mitral valve. Still images and video were sent to a cardiologist for review. Rapid progression of disease in ZF3 prohibited scheduling of a comprehensive echocardiogram by a cardiologist.

Infants ZF4 (PD 1, PD 17) and ZF5 (PD 0, PD 16) underwent comprehensive two dimensional and Doppler echocardiography (Sonos 5500, Philips Ultrasound, Andover, MA) with a high-frequency phased-array probe to assess chamber dimensions, ventricular function, and valve function according to guidelines published by the American Society of Echocardiography [92, 93]. Specific assessment for common congenital heart disease including atrial and ventricular septal defect, transposition of the great arteries, truncus arteriosus, patent ductus arteriosus, and valvular and ventricular atresia was performed. Infants were awake and swaddled for echocardiograms.

### Electrocardiography

Limb lead only (six-lead) electrocardiograms were performed on three infected infants (ZF3, GD 159; ZF4, GD 149; ZF5, GD 162) and one control (CF3, GD 161). ECGs were recorded in right lateral recumbency while the infant was awake and swaddled. Electrodes were placed at the medial elbows and medial stifles of each limb, and electrocardiographic recordings were made on a CARDIOVIT AT-2 Light (Schiller). Mean recording length was 85 seconds (range 36 to 195 seconds, dependent upon infant temperament). A board certified veterinary cardiologist reviewed the ECGs of infected infant ZF3 and control CF3; all other ECGs were reviewed by a clinical veterinarian.

### Electroencephalogram

Infants ZF4 and ZF5 were bottle fed immediately prior to electroencephalography to induce drowsiness. The lateral temples were shaved, and the skin was cleaned with ethanol. Prep N' Stay (Pharmaceutical Innovations) was then applied to gauze and gently rubbed onto the shaved area. Dynarex snap EKG electrode pads were trimmed to fit and affixed to the shaved area with Duo Striplash Adhesive (Ardell). Parker Spectra 360 Electrode Gel Tube-250 Gram (Parker) was applied to a digital ring electrode which was then secured on the infant’s leg. The animal was positioned on blankets in an incubator and allowed to fall asleep. EEG and ground leads were then connected to an AM-systems preamplifier with alligator clip cables. Signals were amplified with an AM-systems 1800 Differential AC amplifier, with filter settings 10 Hz to 500 Hz. The EEG was displayed on an oscilloscope. Simultaneous video was recorded of the oscilloscope readout and sleeping infant, and was reviewed by R.F.

### Retinal imaging and optical coherence tomography

A Heidelberg Spectralis SD-OCT system with a standard 30° lens was used to acquire volumetric, 37-line 15-degree square scans centered on the fovea. An Optos ultrawide field retinal imagine device (California model), which enables visualization and imaging of the peripheral retina, was used to acquire red free, choroidal, and autofluorescence images. Neonates were sedated with Telazol (1:1 mixture of tiletamine hydrochloride and zolazepam hydrochloride, 5mg/kg IM). Heart rate and peripheral blood oxygen saturation were monitored by pulse oximetry. Rectal temperature was maintained between 37.0°C and 38.0°C using disposable chemical heating pads placed underneath the animal (Infatherm Infant Transport Mattress, Phillips). Animals were positioned prone with the head supported by a chinrest. Prior to image acquisition, the pupils were dilated to a minimum of 8 mm using phenylephrine (2.5%) and tropicamide (1%) eyedrops. Speculums were used to keep the eyelids open, and clear plano contact lenses were inserted centered over the cornea to prevent corneal drying and to improve image quality. Following imaging, the contacts and speculums were removed, and erythromycin ointment was applied to each eye. Animals were then recovered from sedation and returned to their home cages or enclosures. Optical coherence tomography scans were segmented with all 11 layers identified. The thickness of each retinal layer was quantified and compared to breastfed two-week postnatal ONPRC historical control neonates (approximately GD 182). Control neonates were dam-reared as previously described [94], with OCT images obtained as described above except under isoflurane anesthesia.

### Auditory brainstem response testing

Acoustic stimuli were digitally generated, and auditory responses monitored using a National Instruments PXI stimulus generation/data acquisition system consisting of a 24-bit digital DAQ board (PXI-4461), a 16-bit DAQ board (PXI-6221), and the Eaton Peabody Laboratories Cochlear Function Test Suite. To elicit the ABRs, 5-ms tone pip stimuli were generated with 0.5-ms rise/fall times (cosine squared) in alternating polarity at 30/s. Acoustic stimuli were delivered within the ear canal via a custom acoustic assembly comprising two dynamic drivers as sound sources and a miniature electret microphone to measure sound pressure *in situ*. The response from the electrodes were amplified (10,000x), filtered (300 Hz-3 kHz bandpass), and averaged (512 samples at each frequency-level combination). Animals were sedated with Telazol (6 mg/kg IM) and the plane of anesthesia was maintained with Telazol (3 mg/kg IV) and ketamine (5 mg/kg IV). Four subcutaneous needle electrodes were distributed in the following locations: between the forehead brow and the vertex of the skull at the midline; along the mandible ventral to the pinna; in the shoulder; and in the foreleg. Pure tone stimuli were presented at 2, 4, 8, 16 and 26 kHz. Sound level was incremented in 5 dB steps from ~10 dB below threshold to ~25 dB above the threshold with an upper intensity limit of 85 dB SPL. Threshold for ABRs was defined as the lowest stimulus level at which a repeatable response from two of the five waves could be identified above the noise floor in the ABR waveform.

### Distortion product otoacoustic emissions testing

Following sedation and testing for ABR, two primary tones (f1, f2) with a frequency ratio of 1.2 were presented and the emission and surrounding noise floor recorded in the ear canal by the acoustic probe assembly outlined previously. The f2 primary was presented at 1.5, 2, 4, 8, 12, and 16 kHz from 20 to 70 dB SPL incremented in 10 dB steps with the level of the f1 primary being 10 dB greater. Ear-canal sound pressure was amplified and digitally sampled, fast Fourier transforms were computed and averaged, and the 2f1-f2 DPOAE and surrounding noise floor were extracted. Otoacoustic emissions stimulated by an f2 level of 60 dB SPL and ambient noise floor are presented at each frequency for comparison purposes.

### Postmortem magnetic resonance image acquisition and image processing

Postmortem brains immersed in 0.01M PBS underwent anatomical examinations on a Siemens 3T Tim Trio system. A customized pediatric rhesus head 8-channel volumetric array coil (Rapid MR International, Columbus, OH, USA) and the “body” RF coil was used for signal receiving and transmission, respectively. A three-dimensional (3D) T1-weighted magnetization-prepared rapid gradient-echo (MP-RAGE) was used to acquire T1-weighted (T1W) images at 0.5mm isotropic resolution with TR/TE/TI of 2500/3.9/1100ms and flip angle of 12°. In-plane image sampling consisted of 216 and 256 data points in the phase-encode and readout directions, respectively. SPACE, a 3D fast spin-echo sequence was used to acquire T2-weighted (T2W) images at 0.5mm isotropic resolution with TR/TE of 3200/394 ms, flip angle of 120°, and echo train length of 105. In-plane image sampling consisted of 256 data points along phase-encoding and readout directions and 176 slices were acquired. Two repetitions were acquired for both MPRAGE and SPACE sequences. They were registered together using “flirt” function in FSL toolbox [95–97] and then averaged to increase signal-to-noise ratio (SNR). After averaging, T1W and T2W images were manually masked using ITK_SNAP [98], corrected for bias field using “N4BiasFieldCorrection” tool in ANTS [99], and aligned to a common space [100] using rigid-body transformation for side-by-side comparison. Cortical surface area and nondimensionalized mean curvature (K*) were calculated as previously described [101, 102].

## Supporting information

S1 Fig Maternal and infant humoral response to ZIKV infection. (A) Maternal anti-ZIKV Ab (IgG/M/A) from serial plasma samples. (B) Infant anti-ZIKV Ab (IgG/M/A) from plasma at delivery and/or necropsy. The limit of detection was a dilution = 1:50.
(TIF)

S2 Fig Maternal CD8+ T cell response. Maternal T cells responses were monitored during the course of pregnancy for ZD1-3. PBMCs were isolated from peripheral blood and stained with antibodies specific for (A) proliferating CD8+ T cells and (B) activated CD8+ T cells. Average cell percentages were compared between ZIKV-infected dams (ZD1-3, red line) and control dams (CD1-3, grey line). T cell responses in ZD4-5 were not assessed.
(TIF)

S3 Fig Vitals and supplemental oxygen needs. (A) Fractionally inspired oxygen (FiO2) during resuscitation. Solid lines indicate positive pressure ventilation or continuous positive airway pressure with blended oxygen, or unsupported respirations (room air). Dotted lines indicate flow-by blended oxygen supplementation (imprecise FiO2). (B) Peripheral capillary oxygen saturation (SpO2) during resuscitation. (C) Modified Apgar score at 1, 5 and 30 minutes following delivery. (D) Heart rate during the first 48 hours following delivery. (E) Respiratory rate from delivery to postnatal day 18. Gestational day (GD) listed in legend is at birth.
(TIF)

S4 Fig Necropsy weights and measures. (A) Crown-rump length. (B) Head circumference. (C) Bitemporal diameter. (D) Body weight. (E) Brain weight. (F) Heart weight. (G) Brain weight normalized to crown-rump length. (H) Heart weight normalized to crown-rump length. Dotted lines indicate a 95% prediction interval from historical control data from the Oregon National Primate Research Center (ONPRC). Blue curve delineates 2 s.d. from mean of control data from Kerr *et al*. [47]. Gestational age of ONPRC historical controls (n = 63) was estimated from maternal estradiol levels (n = 56), first trimester ultrasound biometrics (n = 4) or date of embryo transfer (n = 3). Three early-gestation chimeric fetuses (GD 51–56) [88] and two term neonates (GD ~169, 1 day postnatal) are included in control data.
(TIF)

S5 Fig Seizures and management in infant ZF3. Frequency of observed seizures (n = 101, left axis) from birth to euthanasia, plotted alongside administered antiepileptic drug dosages (right axis).
(TIF)

S6 Fig Neonatal developmental assessments. (A) Attainment of motor development criteria by gestational age. (B) Attainment of survival reflex criteria by gestational age. (C) Attainment of motor development criteria by postnatal age. (D) Attainment of survival reflex criteria by postnatal age. Filled shapes indicate the age at which a criterion was achieved. Unfilled shapes, graphed at the last gestational or postnatal day of testing, indicate a neonate did not meet a criterion prior to euthanasia.
(TIF)

S7 Fig Postnatal growth. Body weight change as a percent of birth weight PD 0–18. Failure to thrive in ZF3 was attributed to feeding difficulties including difficulty swallowing. Declining weight in this infant coincided with the onset of cardiorespiratory symptoms on PD 10.
(TIF)

S8 Fig Normal retinal imaging in ZF4 and ZF5. **(A, B)** Representative in vivo ultra-widefield and (C, D) optical coherence tomography (OCT) images of the right eye of infant ZF4. Panel A illustrates a green-free image and B illustrates a red-free image. Panel C is an OCT image of the right eye of ZF4. Segmenting lines have been drawn on all layers of the retina in panel D. (E) Retinal layer thickness in ZF4 and ZF5 compared against average layer thickness in 2-week postnatal ONPRC historical controls (n = 8). No lesions or abnormalities in retinal layer thickness were detected in ZF4 or ZF5. ILM = Internal Limiting Membrane; RNFL = Retinal Nerve Fiber Layer; GCL = Ganglion Cell Layer; IPL = Inner Plexiform Layer; INL = Inner Nuclear Layer; OPL = Outer Plexiform Layer; ONL = Outer Nuclear Layer; IS/OS = Junction of Inner and Outer Photorecepter Segments; RPE = Retinal Pigment Epithelium; BM = Bruch’s Membrane.
(TIF)

S9 Fig Auditory function and cochlear hair cell sparing in ZF4 and ZF5. (A) ABR thresholds in neonates exposed to ZIKV *in utero*. Infant ZF5 demonstrated thresholds consistent with 1-month-old controls. ZF4 did not respond to sound at 2 and 8 kHz (arrows indicate absence of responses below the test limit) and presented elevated thresholds at 12, 16, and 26 kHz. (B) DPOAE responses in neonates exposed to ZIKV *in utero*. ZF5 responded at all frequencies tested except 4 kHz, consistent with controls. ZF4 responded minimally at 12 kHz, with no response at the other frequencies tested. The dashed lines indicate the noise floor for animal or group. The stimulus level of f2 was 60 dB SPL. (C) Whole mount immunofluorescence for the hair cell marker myosin VIIA (green) and neurofilament (pink) along the middle turn of ZF4. Neurofilament positive radial fiber (RF) distribution is consistent with normal innervation. (D) Hair cell and neurofilament distribution along the tonotopic axis of ZF4. (E) Inner and outer hair cell quantification along the tonotopic axis of the cochlea in ZF4 and ZF5. The number of myosin VIIA positive inner and outer hair cells per 10 μm segment of basilar membrane were consistent along the apical to basal axis of the cochlea. The variability in the number of outer hair cells in the most apical regions was associated with an additional row of outer hair cells. Heterogeneity in outer hair cell row number is common in the apical cochlea. Scale bars = 100 μm. Control infants (n = 3) were born at term (approximately GD 168), dam-reared, and tested on PD 34–42 (approximately GD 202–210).
(TIF)

S1 Table Reduced Purkinje cell numbers in cerebellums of ZIKV-infected infants. H&E stained central cerebellar folia from four gyri, four sulci and four sides from each case were examined at 400x. Purkinje cells with visible nuclei were counted in each field of view. Purkinje cell numbers in ZF3-5 were compared to counts in CF3. Purkinje cell numbers in ZF1-2 were compared to a GD 127 historical control.
(TIF)
