# Supplementary figures and images for: A neonatal nonhuman primate model of gestational Zika virus infection with evidence of microencephaly, seizures and cardiomyopathy
Source: PLoS One. 2020 Jan 14;15(1):e0227676. doi: 10.1371/journal.pone.0227676 (PMC6959612; doi:10.1371/journal.pone.0227676)

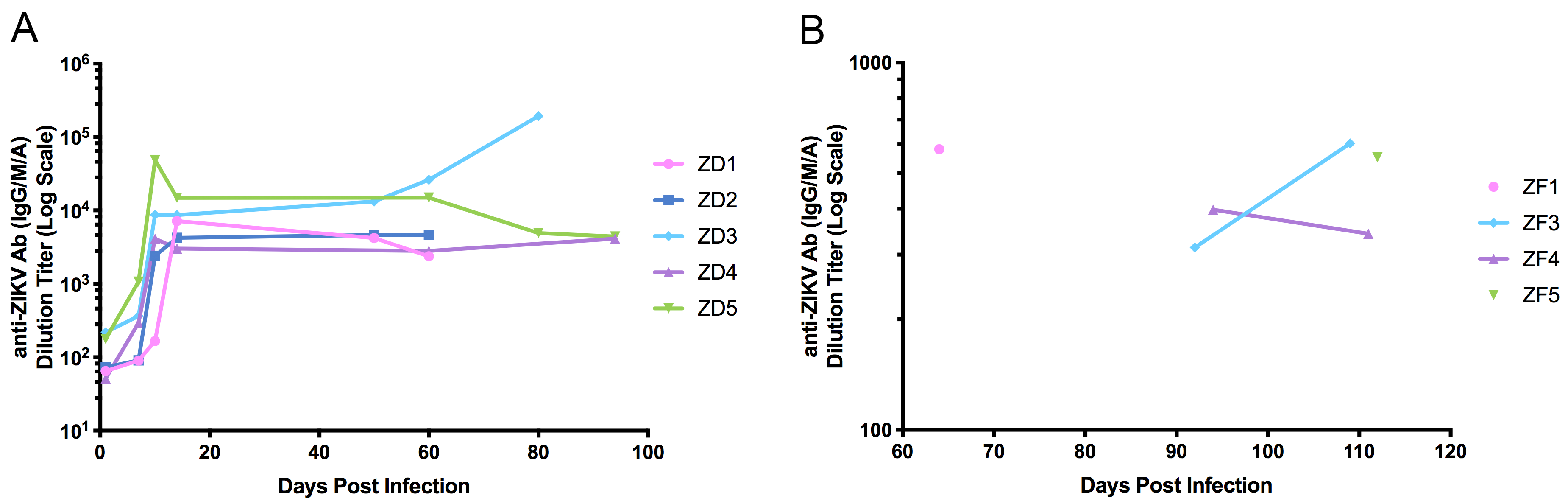

Supplement: S1 Fig — (A) Maternal anti-ZIKV Ab (IgG/M/A) from serial plasma samples. (B) Infant anti-ZIKV Ab (IgG/M/A) from plasma at delivery and/or necropsy. The limit of detection was a dilution = 1:50. (TIF) [file pone.0227676.s001.tif]

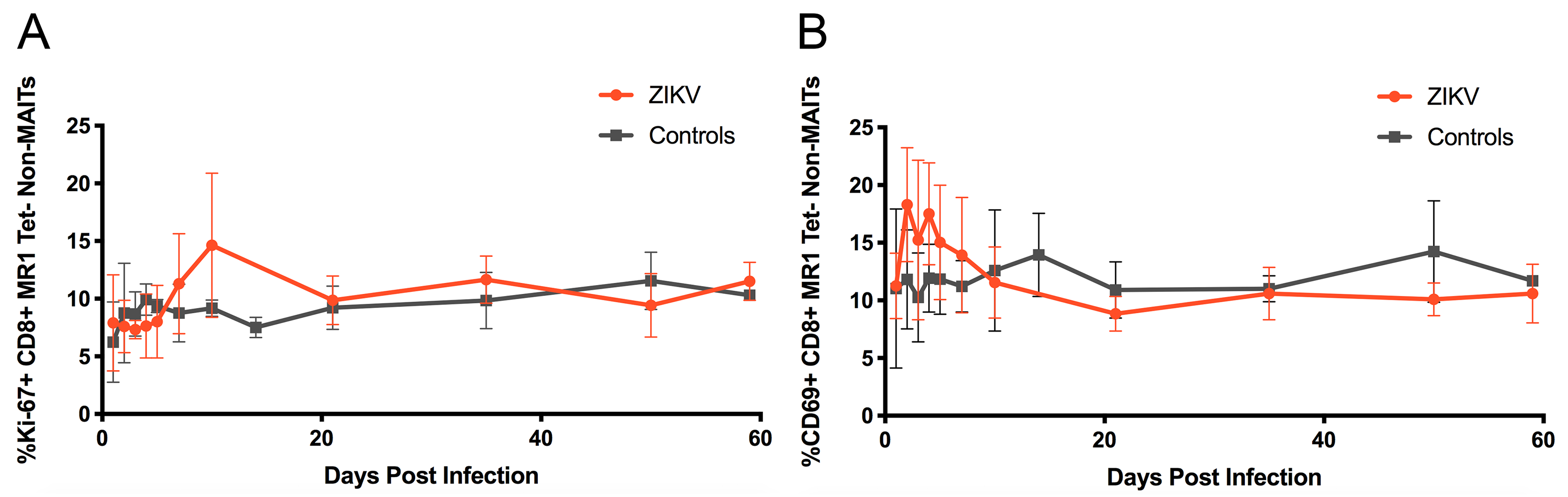

Supplement: S2 Fig — Maternal T cells responses were monitored during the course of pregnancy for ZD1-3. PBMCs were isolated from peripheral blood and stained with antibodies specific for (A) proliferating CD8+ T cells and (B) activated CD8+ T cells. Average cell percentages were compared between ZIKV-infected dams (ZD1-3, red line) and control dams (CD1-3, grey line). T cell responses in ZD4-5 were not assessed. (TIF) [file pone.0227676.s002.tif]

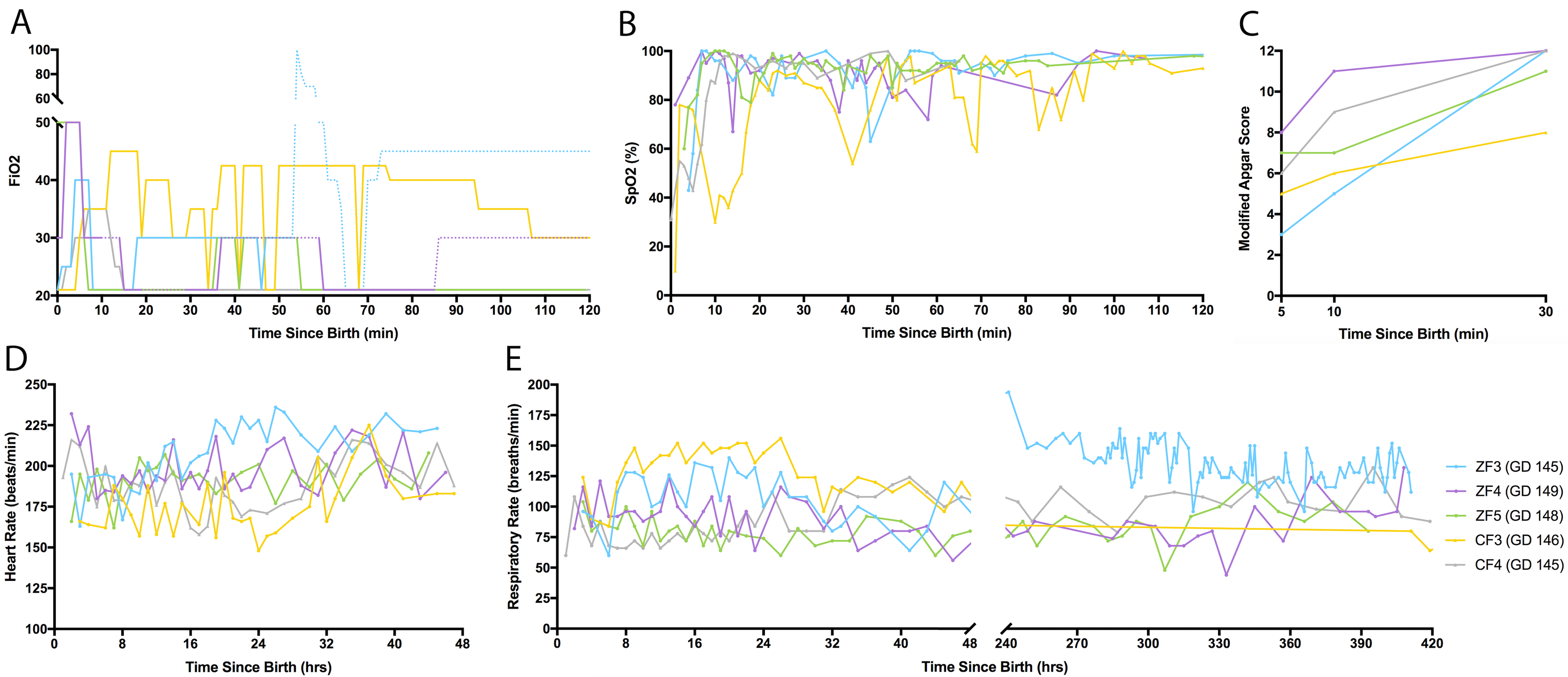

Supplement: S3 Fig — (A) Fractionally inspired oxygen (FiO2) during resuscitation. Solid lines indicate positive pressure ventilation or continuous positive airway pressure with blended oxygen, or unsupported respirations (room air). Dotted lines indicate flow-by blended oxygen supplementation (imprecise FiO2). (B) Peripheral capillary oxygen saturation (SpO2) during resuscitation. (C) Modified Apgar score at 1, 5 and 30 minutes following delivery. (D) Heart rate during the first 48 hours following delivery. (E) Respiratory rate from delivery to postnatal day 18. Gestational day (GD) listed in legend is at birth. (TIF) [file pone.0227676.s003.tif]

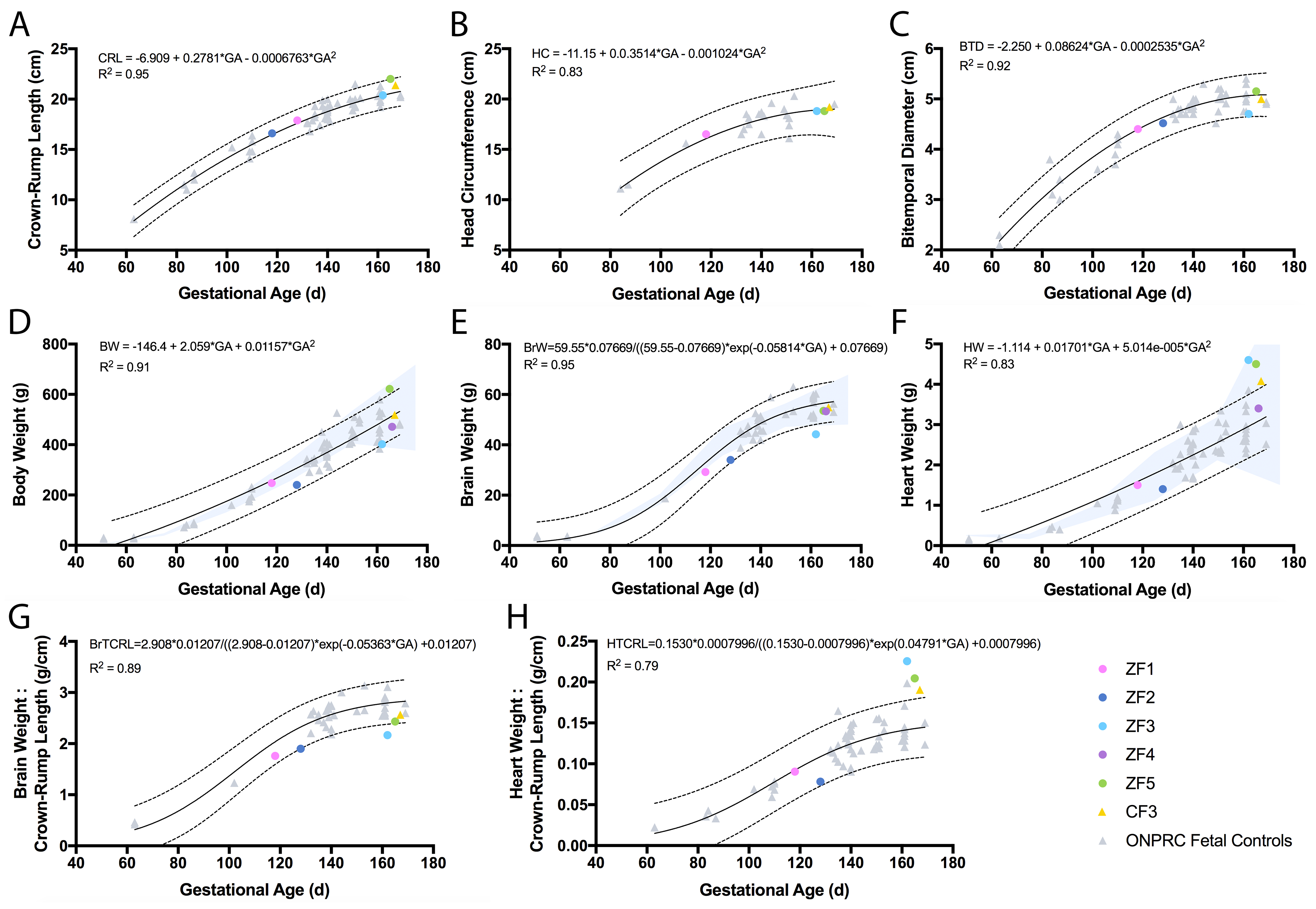

Supplement: S4 Fig — (A) Crown-rump length. (B) Head circumference. (C) Bitemporal diameter. (D) Body weight. (E) Brain weight. (F) Heart weight. (G) Brain weight normalized to crown-rump length. (H) Heart weight normalized to crown-rump length. Dotted lines indicate a 95% prediction interval from historical control data from the Oregon National Primate Research Center (ONPRC). Blue curve delineates 2 s.d. from mean of control data from Kerr et al. [47]. Gestational age of ONPRC historical controls (n = 63) was estimated from maternal estradiol levels (n = 56), first trimester ultrasound biometrics (n = 4) or date of embryo transfer (n = 3). Three early-gestation chimeric fetuses (GD 51–56) [88] and two term neonates (GD ~169, 1 day postnatal) are included in control data. (TIF) [file pone.0227676.s004.tif]

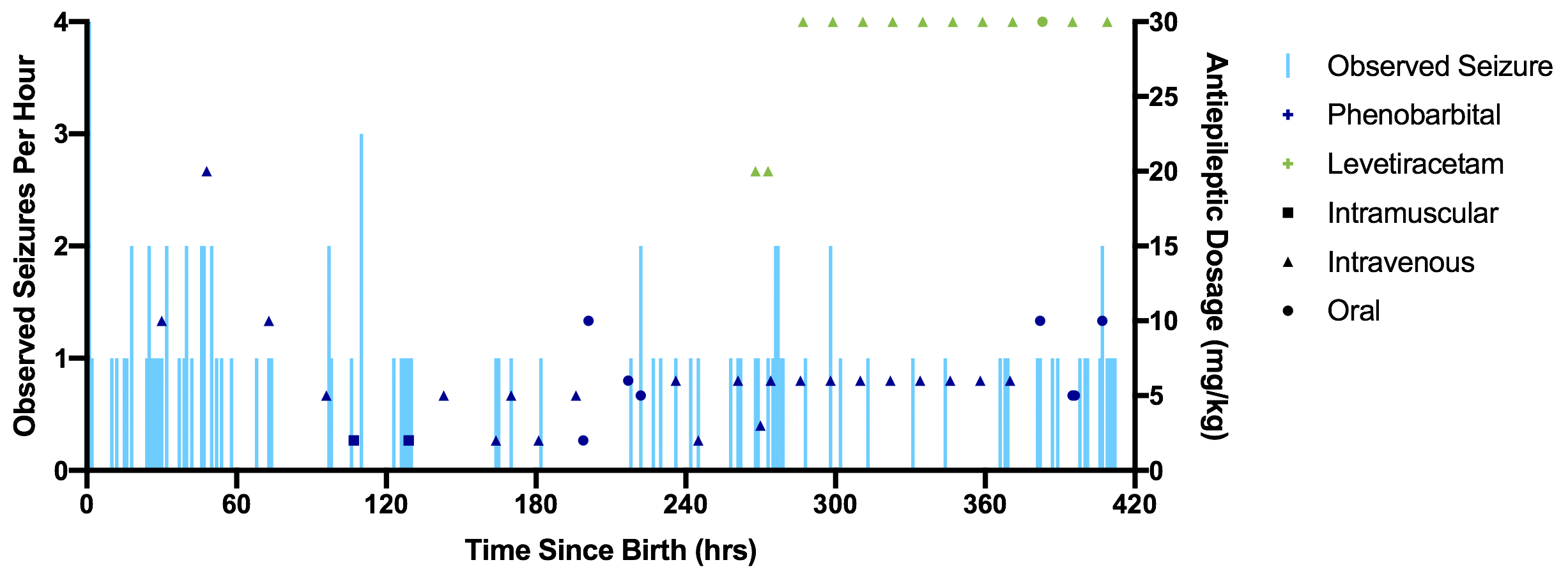

Supplement: S5 Fig — Frequency of observed seizures (n = 101, left axis) from birth to euthanasia, plotted alongside administered antiepileptic drug dosages (right axis). (TIF) [file pone.0227676.s005.tif]

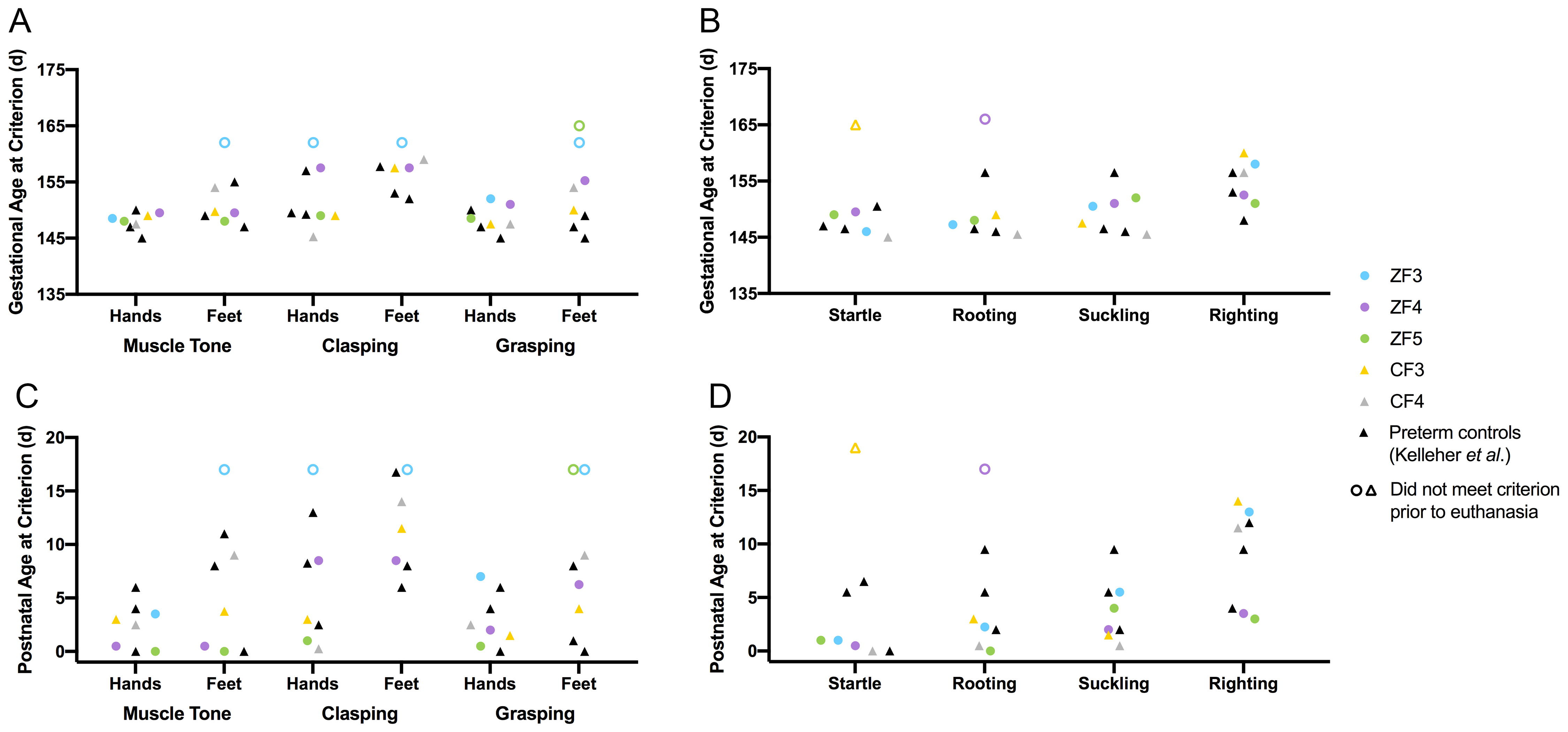

Supplement: S6 Fig — (A) Attainment of motor development criteria by gestational age. (B) Attainment of survival reflex criteria by gestational age. (C) Attainment of motor development criteria by postnatal age. (D) Attainment of survival reflex criteria by postnatal age. Filled shapes indicate the age at which a criterion was achieved. Unfilled shapes, graphed at the last gestational or postnatal day of testing, indicate a neonate did not meet a criterion prior to euthanasia. (TIF) [file pone.0227676.s006.tif]

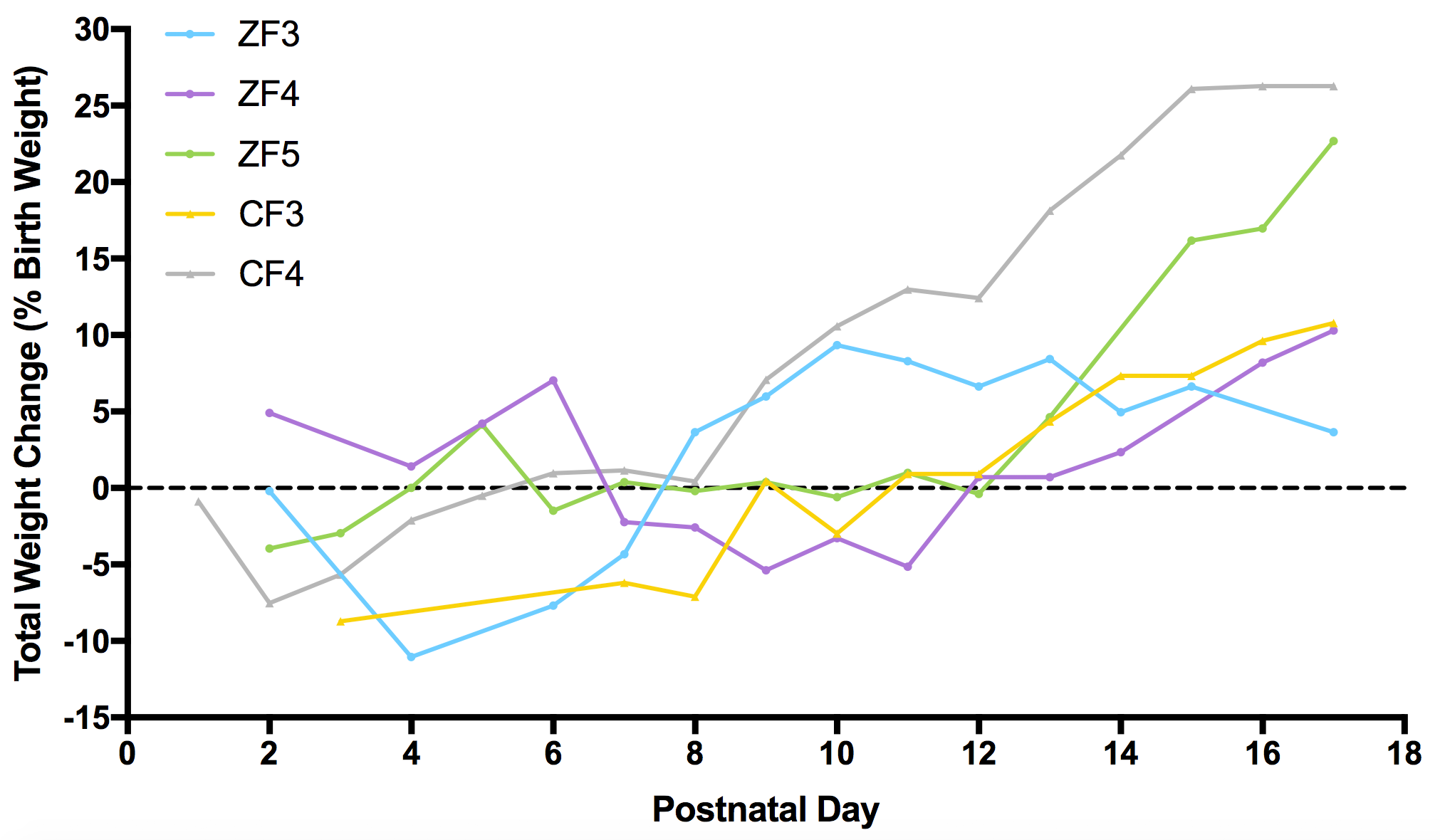

Supplement: S7 Fig — Body weight change as a percent of birth weight PD 0–18. Failure to thrive in ZF3 was attributed to feeding difficulties including difficulty swallowing. Declining weight in this infant coincided with the onset of cardiorespiratory symptoms on PD 10. (TIF) [file pone.0227676.s007.tif]

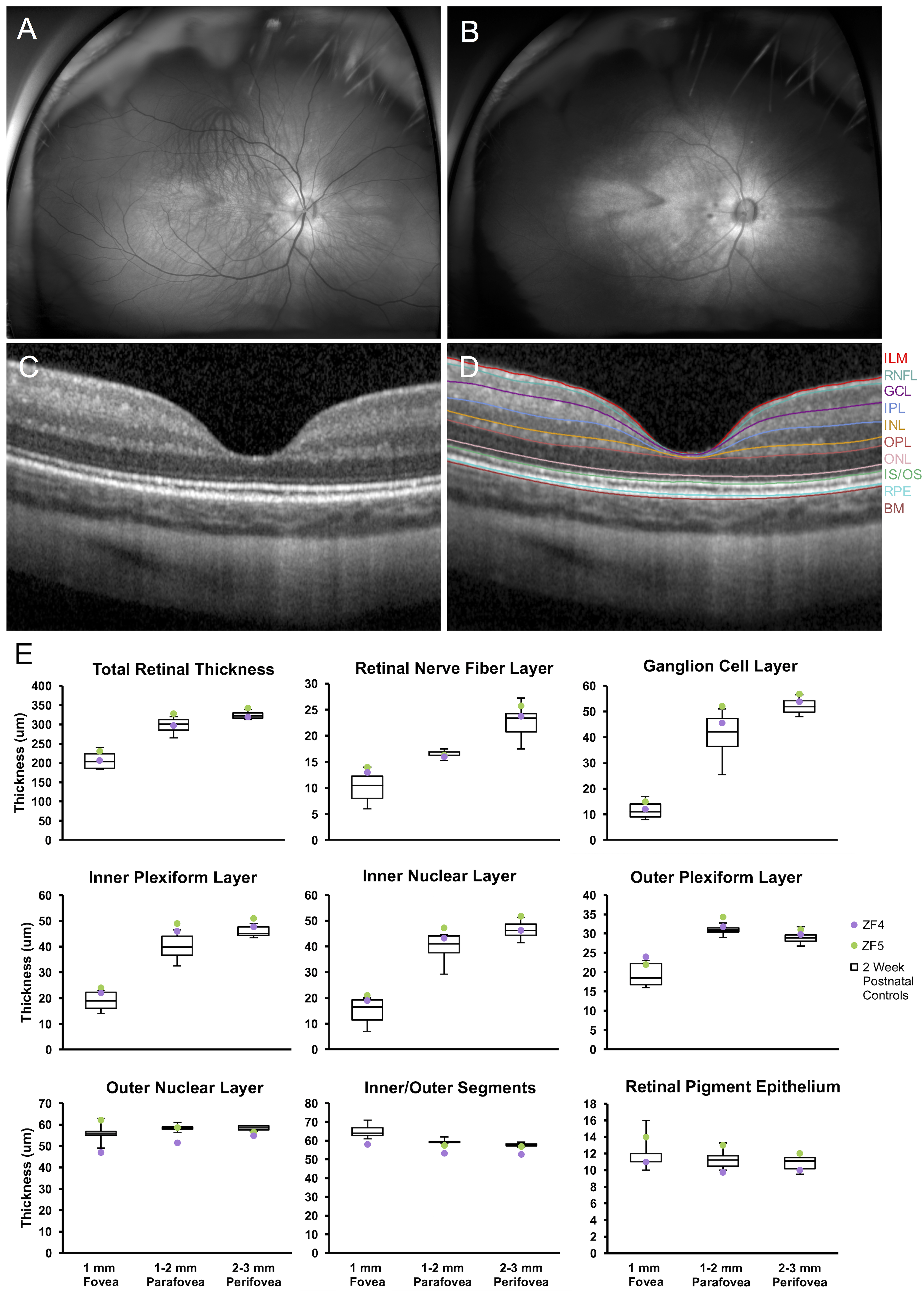

Supplement: S8 Fig — (A, B) Representative in vivo ultra-widefield and (C, D) optical coherence tomography (OCT) images of the right eye of infant ZF4. Panel A illustrates a green-free image and B illustrates a red-free image. Panel C is an OCT image of the right eye of ZF4. Segmenting lines have been drawn on all layers of the retina in panel D. (E) Retinal layer thickness in ZF4 and ZF5 compared against average layer thickness in 2-week postnatal ONPRC historical controls (n = 8). No lesions or abnormalities in retinal layer thickness were detected in ZF4 or ZF5. ILM = Internal Limiting Membrane; RNFL = Retinal Nerve Fiber Layer; GCL = Ganglion Cell Layer; IPL = Inner Plexiform Layer; INL = Inner Nuclear Layer; OPL = Outer Plexiform Layer; ONL = Outer Nuclear Layer; IS/OS = Junction of Inner and Outer Photorecepter Segments; RPE = Retinal Pigment Epithelium; BM = Bruch’s Membrane. (TIF) [file pone.0227676.s008.tif]

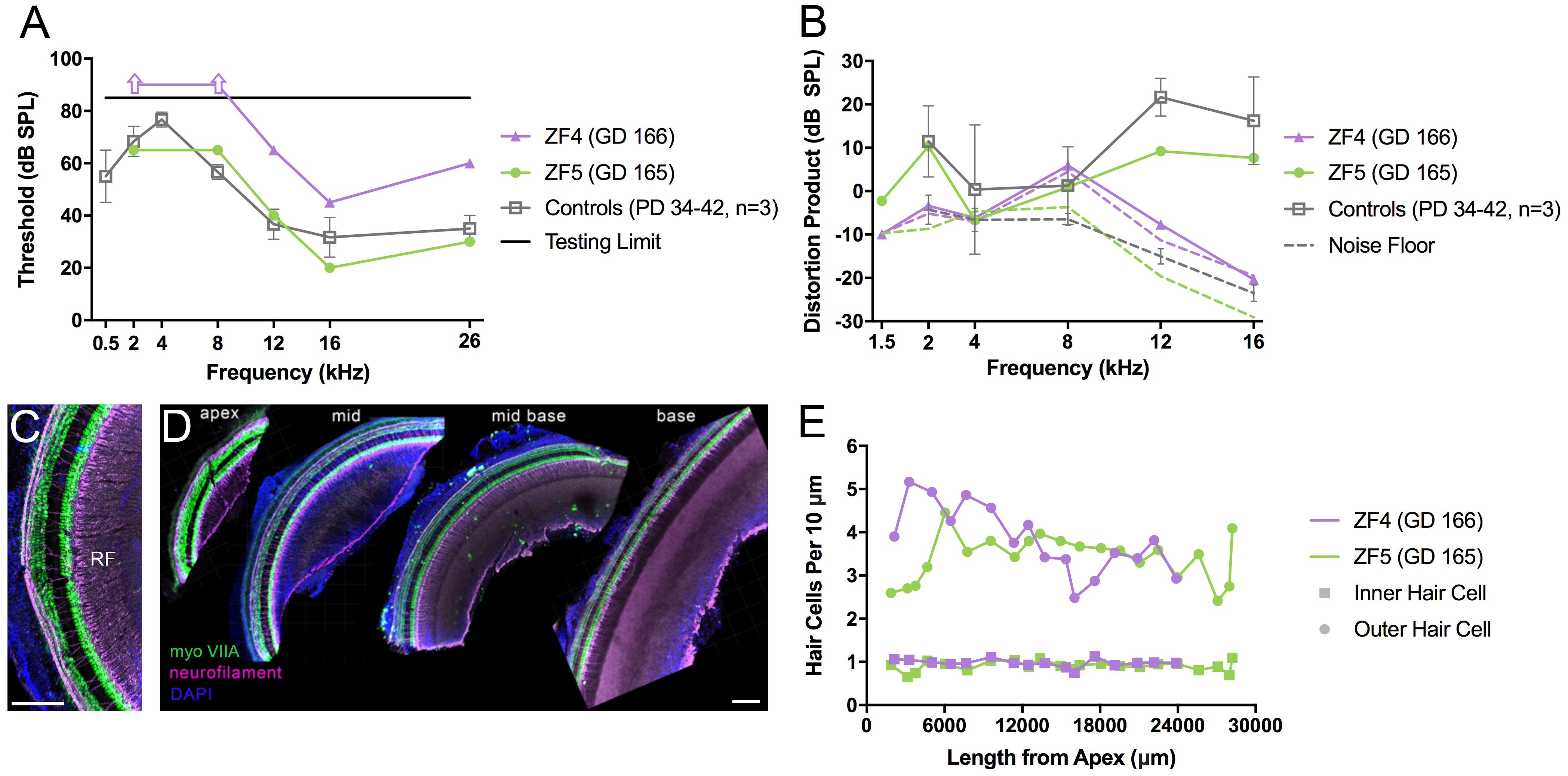

Supplement: S9 Fig — (A) ABR thresholds in neonates exposed to ZIKV in utero. Infant ZF5 demonstrated thresholds consistent with 1-month-old controls. ZF4 did not respond to sound at 2 and 8 kHz (arrows indicate absence of responses below the test limit) and presented elevated thresholds at 12, 16, and 26 kHz. (B) DPOAE responses in neonates exposed to ZIKV in utero. ZF5 responded at all frequencies tested except 4 kHz, consistent with controls. ZF4 responded minimally at 12 kHz, with no response at the other frequencies tested. The dashed lines indicate the noise floor for animal or group. The stimulus level of f2 was 60 dB SPL. (C) Whole mount immunofluorescence for the hair cell marker myosin VIIA (green) and neurofilament (pink) along the middle turn of ZF4. Neurofilament positive radial fiber (RF) distribution is consistent with normal innervation. (D) Hair cell and neurofilament distribution along the tonotopic axis of ZF4. (E) Inner and outer hair cell quantification along the tonotopic axis of the cochlea in ZF4 and ZF5. The number of myosin VIIA positive inner and outer hair cells per 10 μm segment of basilar membrane were consistent along the apical to basal axis of the cochlea. The variability in the number of outer hair cells in the most apical regions was associated with an additional row of outer hair cells. Heterogeneity in outer hair cell row number is common in the apical cochlea. Scale bars = 100 μm. Control infants (n = 3) were born at term (approximately GD 168), dam-reared, and tested on PD 34–42 (approximately GD 202–210). (TIF) [file pone.0227676.s009.tif]

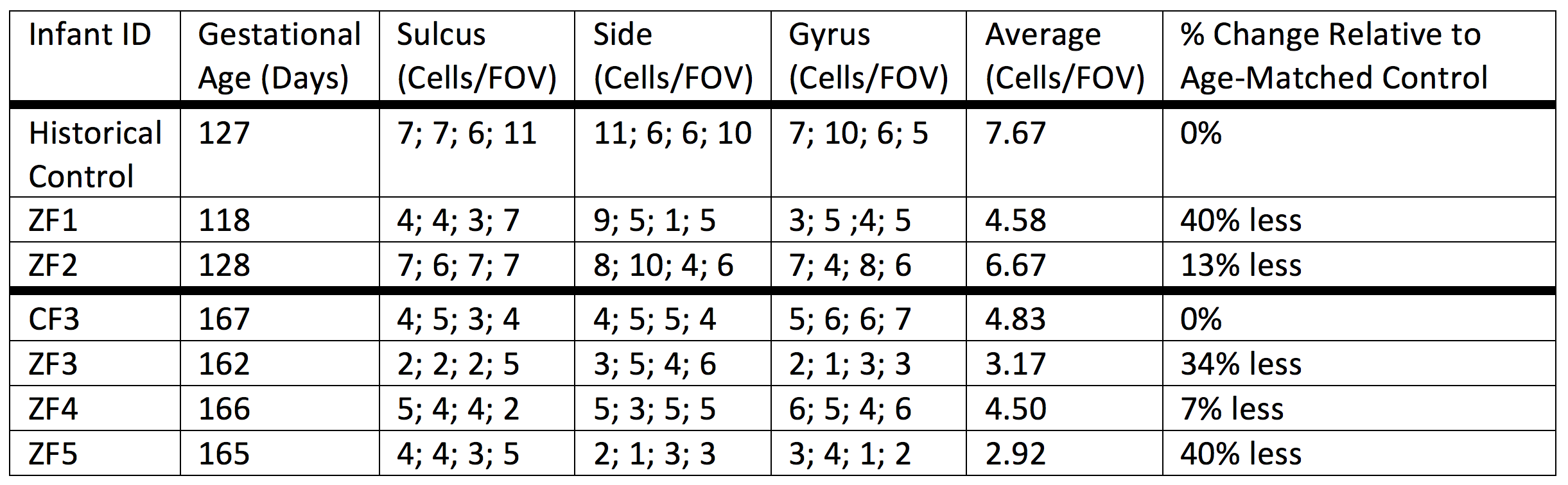

Supplement: S1 Table — H&E stained central cerebellar folia from four gyri, four sulci and four sides from each case were examined at 400x. Purkinje cells with visible nuclei were counted in each field of view. Purkinje cell numbers in ZF3-5 were compared to counts in CF3. Purkinje cell numbers in ZF1-2 were compared to a GD 127 historical control. (TIF) [file pone.0227676.s010.tif]
